# Oral administration of antibiotics increased the potential mobility of bacterial resistance genes in the gut of the fish *Piaractus mesopotamicus*

**DOI:** 10.1186/s40168-019-0632-7

**Published:** 2019-02-18

**Authors:** Johan S. Sáenz, Tamires Valim Marques, Rafael Simões Coelho Barone, José Eurico Possebon Cyrino, Susanne Kublik, Joseph Nesme, Michael Schloter, Susanne Rath, Gisle Vestergaard

**Affiliations:** 1 Comparative Microbiome Analysis, Helmholtz Zentrum München, Neuherberg, 85764 Germany; 2 Institute of Chemistry, University of Campinas, Campinas, Brazil; 3 Section of Microbiology, Department of Biology, University of Copenhagen, 2100 Copenhagen, Denmark; 4 Departamento de Zootecnia, Escola Superior de Agricultura Luiz de Queiroz, University of São Paulo, Piracicaba, Brazil; 5 ZIEL - Institute for Food & Health, Technical University of Munich, 85354 Freising, Germany

**Keywords:** Metagenome, Antibiotic resistance genes, Mobile genetic elements, Gut microbiome, *Piaractus mesopotamicus*, Florfenicol

## Abstract

**Background:**

Aquaculture is on the rise worldwide, and the use of antibiotics is fostering higher production intensity. However, recent findings suggest that the use of antibiotics comes at the price of increased antibiotic resistance. Yet, the effect of the oral administration of antibiotics on the mobility of microbial resistance genes in the fish gut is not well understood. In the present study, *Piaractus mesopotamicus* was used as a model to evaluate the effect of the antimicrobial florfenicol on the diversity of the gut microbiome as well as antibiotic resistance genes (ARGs) and mobile genetic elements (MGEs) using a metagenomic approach.

**Results:**

The total relative abundance of ARGs and MGEs significantly increased during the antibiotic exposure. Additionally, phage integrases, transposases, and transposons flanking ARGs accumulated in the gut microbiome of *P. mesopotamicus* because of the antibiotic exposure. MGEs co-occurring with ARGs showed a significant positive correlation with the total ARGs found. Furthermore, shifts in the gut microbiome towards well-known putative pathogens such as *Salmonella*, *Plesiomonas*, and *Citrobacter* were observed following florfenicol treatment. Mainly *Plesiomonas* and *Citrobacter* harbored genes that code for multidrug and phenicol efflux pumps. Moreover, several genes related to RNA processing and modification, cell motility, SOS response, and extracellular structure were enriched due to the antibiotic application. The observed effects were visible during the complete application phase and disappeared at the post-exposure phase.

**Conclusions:**

Our findings suggest that the oral administration of antibiotics increases the potential for MGE-mediated exchange of ARGs in the gut of fish and could contribute to the enrichment and dispersion of ARGs in aquaculture systems. Importantly, this increase in the potential for ARGs exchange could be an effect of changes in community structure and/or ARG mobilization.

**Electronic supplementary material:**

The online version of this article (10.1186/s40168-019-0632-7) contains supplementary material, which is available to authorized users.

## Background

The rapid acquisition of genes coding for antibiotic resistance of bacteria is a major health concern. It has been stated that the continuous increase of pathogenic bacteria which are resistant against commonly used antibiotics will induce in 2050 up to 10 million cases of death per year and global costs of 100 trillion USD [1]. In recent years, especially metagenomic sequencing of samples from various ecosystems has revealed the large size of the antibiotic resistome, which includes both intrinsic and acquired resistance but also phenotypically silent and protoresistance genes [2].

Aquaculture poses a potential risk for the dissemination of antibiotic resistance genes (ARGs) and mobile genetic elements (MGEs) due to the widespread use of antibiotics [3]. The production of diadromous, freshwater, and marine fish increased from 20.8 million tons in 2000 to 51.9 million tons in 2015 [4]. This increase in production was accompanied by the use of multiple antibiotics, some of them labeled as “critically important,” “highly important,” and “important” according to the antimicrobial WHO list [5], although the use of antibiotics in aquaculture shows clear regional patterns.

Diversity and abundance of ARGs and MGEs have been explored in fish feces [6], fishmeal [7], and sediments of sites where aquaculture has been applied [8–11]. Overall, the available data indicate that different genes conferring resistance to oxytetracycline, quinolones, sulfa/trimethoprim, florfenicol, and amoxicillin are closely associated with aquaculture [6–8, 12]. Additionally, several antibiotic-resistant bacterial strains have been isolated from fish and fish farms’ sediments exposed and not exposed to antibiotics [13, 14].

However, the origin and potential spreading of genes that mediate antibiotic resistance in aquaculture is not clear [3]. A study in 2006 reported that the selection of antibiotic resistance in an integrated marine aquaculture system occurred in the intestine of fish rather than in the sediments [15]. Yet, most of the recent studies used water or sediments from aquaculture farms and not directly fish gut samples. Muziasari et al. postulated that the feces from fish grown in aquaculture was a driver for increased ARGs in the sediments of the aquaculture farms [6], indicating that the gut of the animals could be considered as a hotspot for ARGs and MGEs and one probable origin of dispersion.

In the frame of this study, we investigated the influence of florfenicol, a broad-spectrum fluorine derivative of chloramphenicol frequently used in aquaculture [5], on the composition, function, and distribution of ARGs and MGEs in the gut microbiome of *Piaractus mesopotamicus*, a commonly farmed fish in South America. The objectives of the study were (1) to investigate the diversity and abundance of ARGs and MGEs before, during, and after antibiotic exposure, (2) to evaluate the co-occurrence of MGEs and ARGs and (3) to link ARGs to their respective host bacteria.

## Results

We analyzed the consequences of the oral administration of the antibiotic florfenicol on the bacterial diversity and ARGs and MGEs composition in the intestines of *P. mesopotamicus* during a time series experiment of 34 days including pre-exposure phase, exposure phase, and post-exposure phase. The obtained data was compared to control animals, which did not receive antibiotics but were kept under the same conditions. As expected, the body weight of the sampled fish slightly increased over the experimental period from 651.4 ± 107.1 g at day 0 to 766.2 ± 165.8 g at the end of the antibiotic exposure phase and 781.44 ± 171.1 g at the post-exposure phase. An effect of the antibiotic treatment at the end of the exposure phase comparing treated and control animals was not visible (Additional file 1: Figure S1).

### Reads quality and general annotation

Sequencing produced between 0.8 and 3.4 million paired-end reads per sample. Reads with low quality and sequences considered as contaminants (host DNA or PhiX) were removed (0.006–24.59% of all reads). Clean reads were taxonomically annotated using Kaiju: 25.10–94.02% corresponded to *Bacteria*, 0.48–9.22% *Eukaryota*, 0.01–1.05% *Archaea*, and 0.02–0.89% *Virus*. Only bacterial reads were functionally annotated; between 35.93–44.81% could be annotated using the eggNOG and COG database and Diamond with *e* values below 0.001. The coverage of the metagenome for bacterial reads was above 70% for all the samples (Additional file 1: Figure S2a). Further, total clean reads were assembled, and between 7112 and 116,988 contigs larger than 500 bp were obtained per sample. N50 was between 536 and 10,913 bp and the maximum length between 8 and 117 kb of the total contigs obtained.

### Shifts in bacterial diversity and phage abundance as a result of antibiotic exposure

**Fig. 1:**
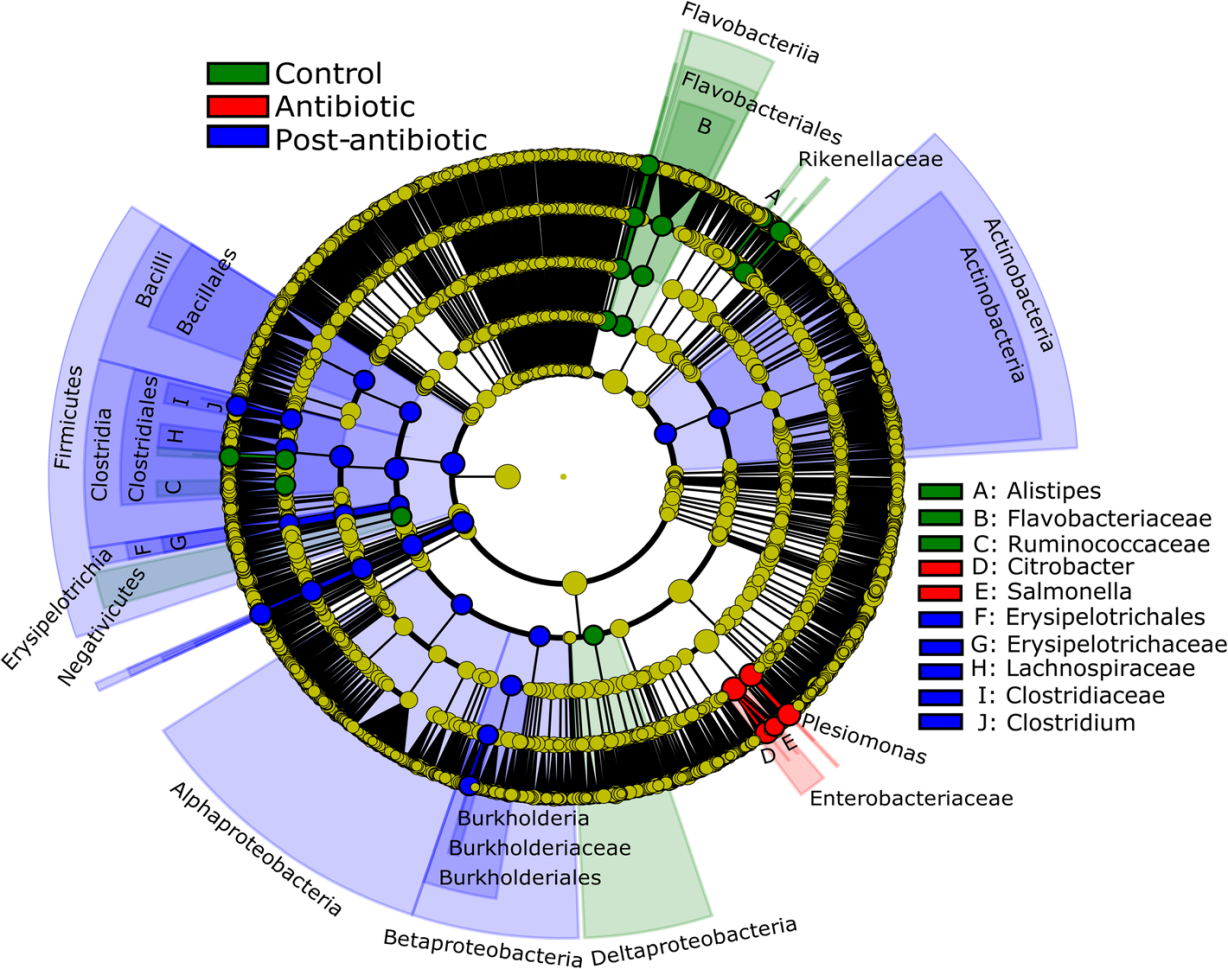
Main bacterial responders of the *P. mesopotamicus* gut microbiome to antibiotic exposure. Cladogram representing the bacterial biomarkers associated to the categories: control/pre-exposure (day 0 and control), antibiotic/exposure (days 1, 4, and 7), and post-antibiotic/post-exposure (days 11, 13, 18, 26, and 34). The size of each circle is proportional to the abundance of the taxon. Colored taxa without label were classified as unassigned. Biomarkers were detected using the LDA effect size (LEfSe). LDA > 3.5, *P* < 0.05

The input of antibiotic did not clearly change metagenomic diversity but altered the abundance of bacterial families. The Nonpareil diversity index oscillated through time, between 13.7 ± 1.5 (min value day 11) and 15.4 ± 0.2 (max value day 18), without a clear influence of the antibiotic treatment (Additional file 1: Figure S2b). Bacterial diversity in the gut of *P. mesopotamicus* was dominated by the families *Bacteroidaceae* (45.7%), *Porphyromonadaceae* (11.2%), *Prevotellaceae* (5.2%), and *Lachnospiraceae* (2.9%) in the pre-exposure phase and was comparable to the control animals, which were kept for the entire experimental period without antibiotic. As a result of the antibiotic exposure, *Bacteroidaceae* were reduced in the gut of the animals, decreasing from 45.7 ± 4.7% at day 0 to 0.02 ± 0.01% at day 7. At the same time, *Enterobacteriaceae* increased from 0.16 ± 0.05% to 53.19 ± 24.3% (Additional file 1: Figure S3a), becoming the most dominant family. Main responders to the antibiotic treatment were the genera *Plesiomonas*, *Salmonella*, and *Citrobacter* (Fig. 1,  *P* < 0.05, LDS > 3.5). Interestingly, also the abundance of phages increased as a result of the antibiotic treatment, from 0.008 to 0.010%, 0.031%, and 0.220% at days 0, 1, 4, and 7 respectively. Aeromonaphages (0.066%), Pseudomonaphages (0.038%), Vibriophages (0.029%), Escherichiaphages (0.018%), Enterobacteriaphages (0.018%), and Salmonellaphages (0.005%) were the most abundant bacteriophage families during exposure with antibiotics at day 7. At day 13 (3 days after the last day of antibiotic exposure), dominant members of the bacterial community structure seem to have recovered. However, differences on the taxonomic structure between day 0 and the post-antibiotic phase were significant (Bray Curtis distance, *P* = 0.001, Adonis) (Additional file 1: Figure S4a). The abundance of phages decreased immediately after the last day of antibiotic exposure and was not different from the pre-exposure phase.

### Functional shifts of the gut microbiome as a result of antibiotic exposure

**Fig. 2:**
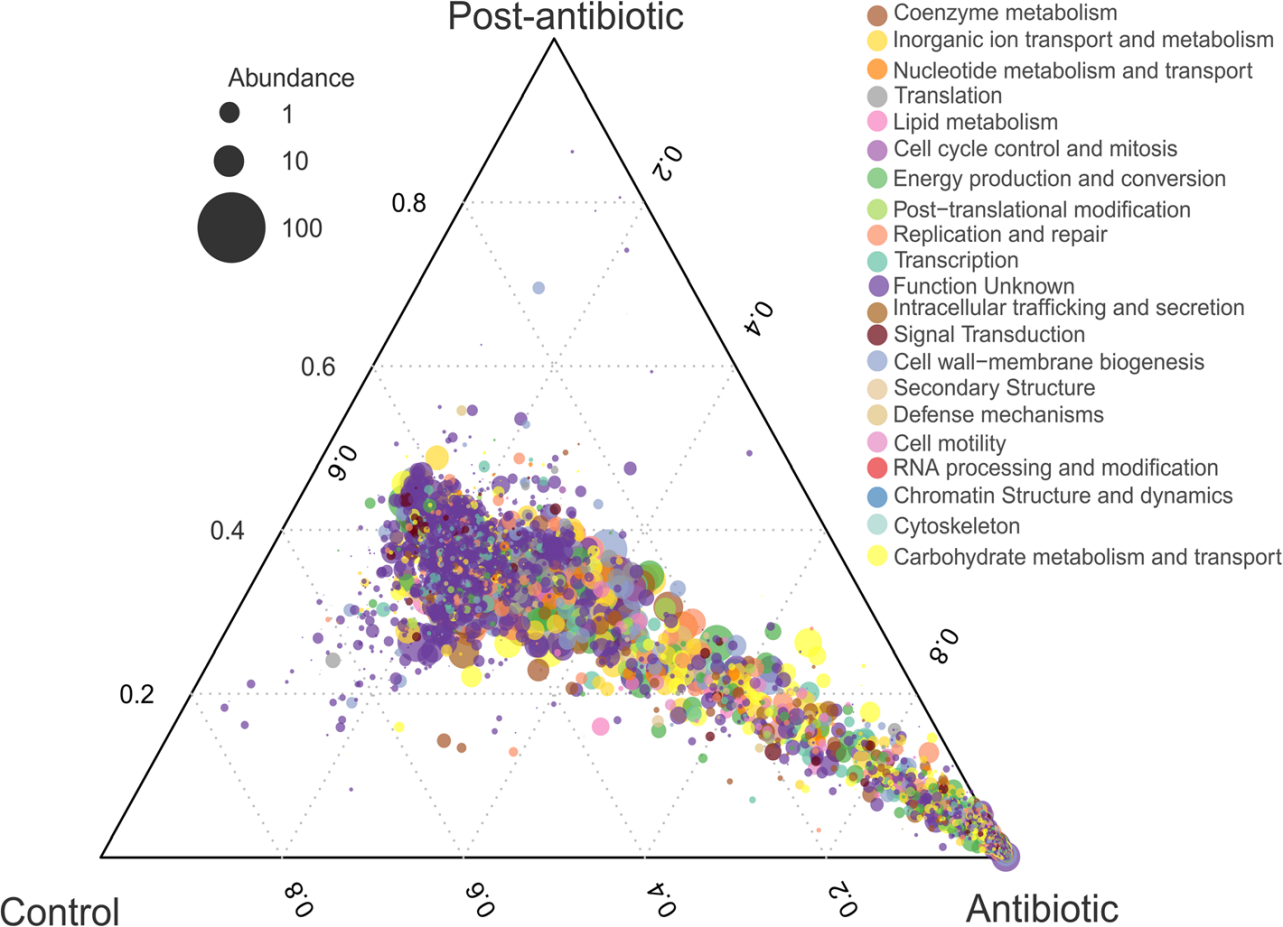
Bacterial functional shift in the gut of *P. mesopotamicus* before, during, and after antibiotic exposure. Ternary plot of the significantly enriched genes between the categories: control/pre-exposure (day 0 and control), antibiotic/exposure (days 1, 4, and 7) and post-antibiotic/post-exposure (days 11, 13, 18, 26, and 34). Significance was detected using the likelihood ratio test (LRT), *P* < 0.05 DeSeq2. Color indicates the categories of Cluster orthologous groups (COGs). The size of plotted dots corresponds to the abundance of the genes represented as the logarithmic scale of the diameter. The dashed grid lines inside the plot designate the contribution of each category

The shift in bacterial community structure was accompanied by significant changes in the function of the gut microbiome as a result of the application of the antibiotic (Fig. 2, *P* < 0.05 likelihood ratio test (LRT)). Relative abundance of 17 out of 23 functional cluster orthologous groups was significantly affected (Additional file 1: Table S1). Out of these 17 functional groups, 11 showed an increase during the exposure phase with the antibiotic. Genes coding for RNA processing and modification, cell motility, and extracellular structure were increased more than 2.5 times (Log_2_ fold change) during the antibiotic treatment compared to day 0. At the same time, genes coding for cytoskeleton (structural filaments) decreased by 2.5 times due to the antibiotic treatment. However, most pronounced changes were in the abundance of genes triggering the bacterial stress response. We found that two orthologous groups for SOS response *recA/lexA* (COG1974) and *recA* modulation activity (COG2137) significantly increased 5 and 13 times during the antibiotic exposure phase (*P* < 0.05 likelihood ratio test (LRT)) respectively. Genes coding for SOS response rise from 0.018 ± 0.001% at day 0 to 0.091 ± 0.009% at day 7 and *recA* modulation from 0.0008 ± 0.00003% to 0.0118 ± 0.001%. At day 13, 3 days after the antibiotic exposure, main functional patterns of the gut microbiome seem to recover and were comparable to the pre-exposure phase. However, differences in the total functional structure between day 0 and the post-antibiotic phase were significant (Bray Curtis distance, *P* = 0.001, Adonis) (Additional file 1: Figure S4b).

### The response of ARGs to antibiotic exposure

**Fig. 3:**
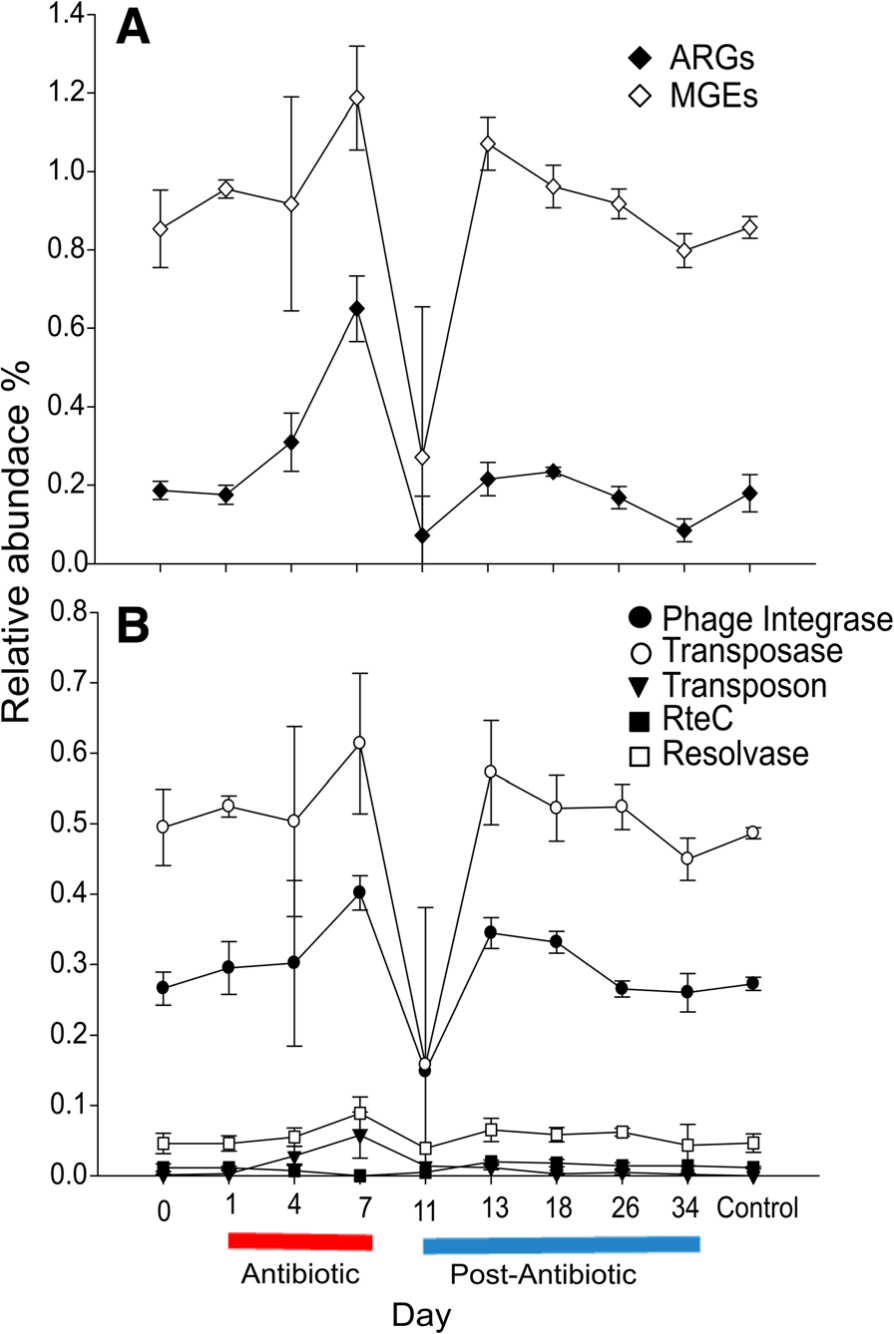
Influence of florfenicol on the relative abundance of total ARGs and MGEs before, during, and after exposure. **a** Relative abundance of total ARGs and MGEs. **b** Relative abundance of groups of MGEs (phage integrases, transposases, transposons, and resolvases). Day 0 (pre-exposure); days 1, 4, and 7 (exposure/florfenicol); and days 11, 13, 18, 26, and 34 (post-exposure). A control sample was taken on day 34 from a tank that did not receive the antibiotic during the whole experiment

Florfenicol application resulted in an increase in the diversity of ARGs and their total relative abundance in the gut microbiome of *P. mesopotamicus* (Fig. 3a). Up to 80 different ARGs were found on days 4 and 7 under antibiotic treatment compared to 16 and 17 ARGs at day 0 and in the control samples respectively. Also, the abundance significantly changed during the different phases (Robust ANOVA, *P* = 0.0009), reaching the maximum value 0.64 ± 0.08% at day 7, compared with 0.18 ± 0.02% at day 0 (Rand Wilcox’s post hoc, *P* = 0.0000). Already at day 0, several ARGs were detected with *mexQ* as the most abundant, followed by *macB*, *mexK*, *acrF*, *rpoB* (*S. aureus*) mutation, and *triC* (Additional file 1: Figure S5a). A similar pattern at day 0 was found in the control sample after 34 days. At day 7, *mexD* was the most abundant ARG followed by *mexQ*, *pmrE*, *macB*, *macA*, and *crp*. In addition, during the exposure phase (days 4, 7) and post-exposure (day 11), the *floR* gene was detected, which codes for resistance against florfenicol. This could indicate an enrichment due to the antibiotic pressure. All the *floR* genes were detected in plasmid sequences, 5 of them belonging to *Proteobacteria*. After the last day of antibiotic exposition, between days 11 and 13, the relative abundance and number of ARGs was comparable to day 0 and the control sample. However, total structure of ARGs was significantly different between the day 0 and days of the post-antibiotic phase being the day 34 the most variable (Bray Curtis distance, *P* = 0.005, Adonis) (Additional file 1: Figure S4C).

**Fig. 4:**
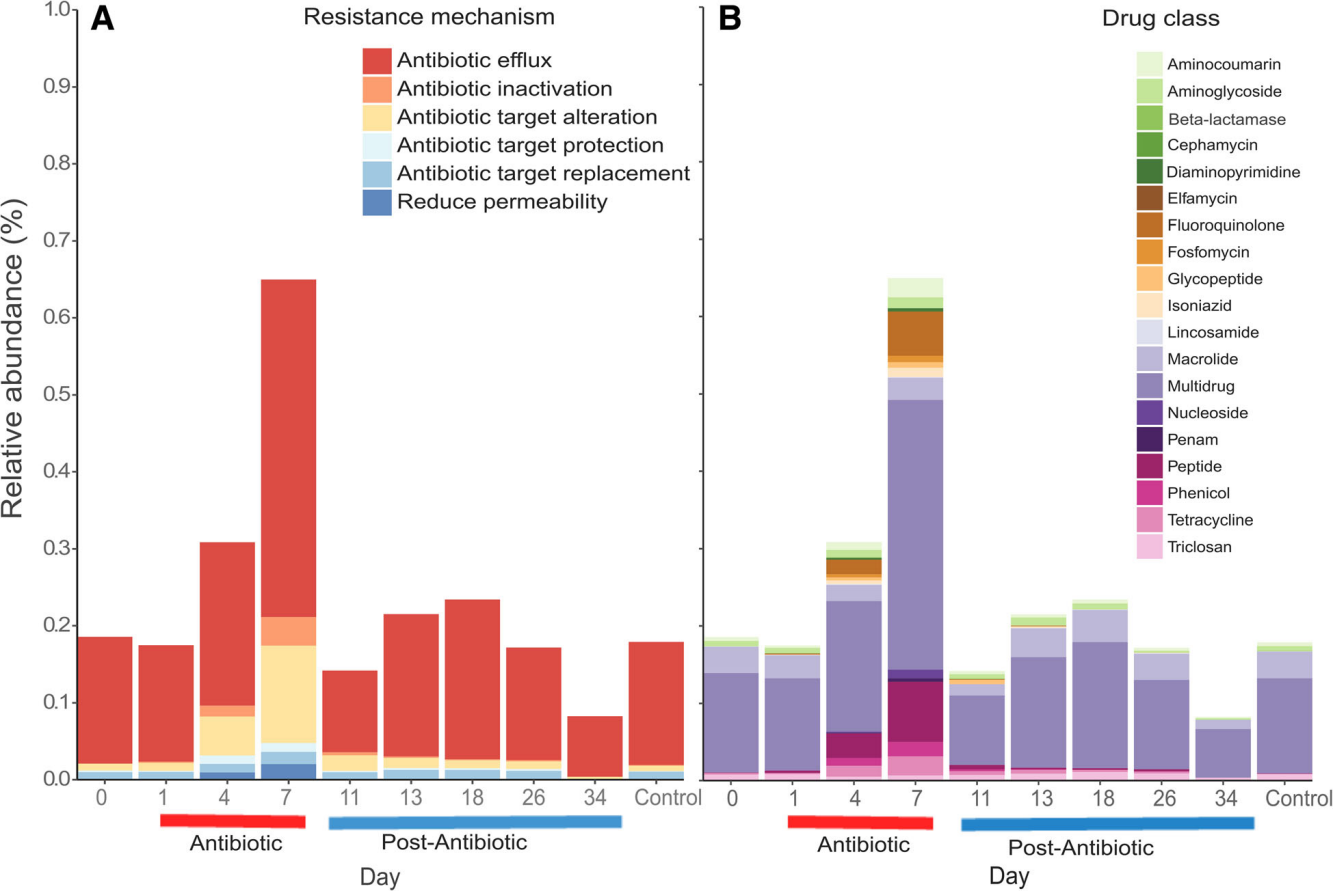
Enrichment of ARGs coding for different drug classes and resistance mechanism during the antibiotic exposure. Changes in the relative abundance of resistance mechanism (**a**) and drug classes (**b**) associated with ARGs detected before, during, and after antibiotic exposure. Day 0 (pre-exposure); days 1, 4, and 7 (exposure/florfenicol); and days 11, 13, 18, 26, and 34 (post-exposure). A control sample was taken on day 34 from a tank that did not receive the antibiotic during the whole experiment

Moreover, genes conferring resistance to eight and nine different drug classes were detected on day 0 and in the control samples, respectively (Fig. 4b). This number increased during the exposure phase, up to 17 different drug classes. Additionally, diversity of drug classes was variable even over the post exposition phase, between 5 and 11. The most abundant genes detected at day 0 corresponded to the drug classes multidrug, macrolide, triclosan, aminoglycoside, and aminocoumarin with relative abundances of 0.126, 0.033, 0.007, 0.007, and 0.004% respectively. At day 7, as a result of the exposure to the antibiotic, the ARGs belonging to the classes multidrug, peptide, fluoroquinolone, aminocoumarin, tetracycline, and phenicol were enriched with relative abundances of 0.349, 0.078, 0.057, 0.024, 0.024, and 0.018% respectively (Rand Wilcox’s post hoc, *P* = 0.0000 all comparisons but fluoroquinolone and phenicol). Peptide drug class was the only one enriched significantly comparing day 0 and the post-antibiotic days 13, 18, and 26 (Rand Wilcox’s post hoc *P* = 0.010, 0.0013, 0 respectively) (Additional file 1: Figure S6). In general, antibiotic efflux was the most important resistance mechanism associated with the ARGs detected (0.078–0.438%) (Fig. 4a). Compared to day 0, the antibiotic treatment increased the genes related to antibiotic efflux, antibiotic target alteration, antibiotic inactivation, and reduced permeability from 0.164 to 0.438, 0.008 to 0.126, 0.0003 to 0.037 and 0 to 0.020% respectively (Rand Wilcox’s post hoc, *P* = 0.0000 all comparisons). After the antibiotic exposure, the abundance of antibiotic efflux genes decreased at day 11 (0.106%) but slightly increased at day 13 (0.173%) after 3 days and 8 days (day 18, 0.207%) of post-exposure phase.

### The response of MGEs to antibiotic exposure

Relative abundance of MGEs (Robust ANOVA, *P* = 0.012) through the different phases was significantly different. The oral application of florfenicol increased the total MGEs in the gut of the animals (Fig. 3a), reaching its highest value at day 7 (1.18 ± 0.13%, compared to day 0 0.853 ± 0.09% and control samples 0.857 ± 0.02%; Fig. 3a) (Rand Wilcox’s post hoc, *P* = 0.0000). This indicates that the input of antibiotics contributes to the enrichment of MGEs in the gut of the animals. The most abundant MGE group identified at day 7 was transposases 0.613%, followed by phage integrases 0.401%, resolvases 0.088%, transposons (Tn3) 0.057%, other 0.025%, and *RteC* 0% (Fig. 3b). However, only for the abundance of phage integrases and transposons, the level of significance was reached when day 7 and the pre-exposure phase were compared (Rand Wilcox’s post hoc, *P* = 0.0000). After day 26, 16 days after the antibiotic exposition, the total abundance of MGEs was comparable to day 0 and control sample.

Between 2.71 ± 0.23 and 8.61 ± 1.96% of the contigs could be classified as a plasmid sequence, with a clear increase as a result of the antibiotic exposure. Of this plasmid sequences, between 0.002 and 0.168% carried ARGs, with the maximum abundance found at the last day of antibiotic exposure (Additional file 1: Figure S7)**.** Most likely, the plasmids were present before and after day 4 and day 7 but in such low abundance that they could not be assembled into contigs. The abundance of contigs carrying ARGs at day 0, post-antibiotic, and control sample was between 0.0026 and 0.0045%, compared to 0.168% at day 7**.**

### Spacial co-occurrence of MGEs and ARGs

**Fig. 5:**
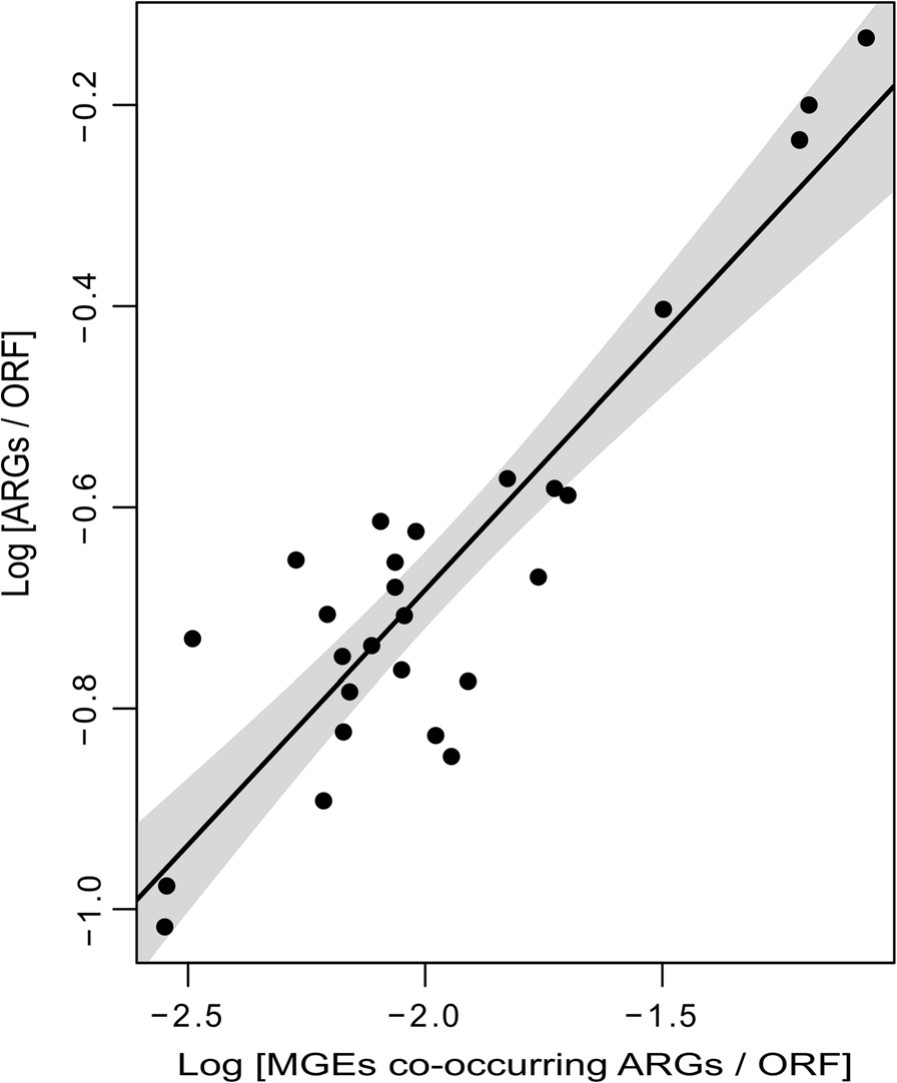
Positive correlation between MGEs co-occurring with ARGs and the total ARGs. Correlation of the log-transformed relative abundance of MGEs co-occurring with ARGs and the total ARGs from the gut of *Piaratus mesopotamicus* before, during, and after the antibiotic exposure. The black line indicates the regression model and the grey area correspond to the 95% confidence interval. Spearman’s correlation = 0.69, *S* = 1006, *P* = 9.4 × 10^−05^. This result was corroborated using a bootstrap Spearman’s correlation (Spearman’s correlation = 0.69, bootstrap = 2000, bias = − 0.011, std. err = 0.14, Percentile interval 95% = 0.341–0.897)

We found a tendency for an increased frequency of co-occurrence between MGEs and ARGs comparing day 0 to 7 (Jonckheere–Terpstra, JT = 48, *P* = 0.0018). Therefore, a positive correlation between ARGs and MGEs co-occurring with ARGs was found (Spearman’s correlation = 0.69, *S* = 1006, *P* = 9.4 × 10^−05^, Fig. 5). This result was corroborated using a bootstrap Spearman’s correlation (bootstrap = 2000, bias = − 0.011, std. err = 0.14, percentile interval 95% = 0.341–0.897). As a control, we calculated the correlation in the co-occurrence between ribosomal proteins L1 and L12 and MGEs (Spearman’s correlation L1—0.24 and L12—0.54) (Additional file 1: Figure S8). Out of all the ARGs detected, 4.19% and 2.41% were found co-occurring with MGEs at day 0 and control sample respectively (Table 1). During the antibiotic exposure phase, the numbers of genes co-occurring increased up to 10.78% at day 7. After the antibiotic exposure, the co-occurrence of genes was around 3.91–5.96% (Table 1). MGEs were co-occurring with 8 different ARGs before the antibiotic exposure, 45 during the antibiotic treatment and 23 during the post-treatment phase. Before the antibiotic exposure, the most abundant gene co-occurring with MGEs was *rpoB* (*S. aureus* mutation, 1.26% of all the ARGs), followed by *gyrA* (*E. coli* mutation), *mexQ*, *mexK*, *and tetQ* (0.56, 0.28, 0.28, and 0.28% respectively) (Additional file 1: Figure S9). During the antibiotic treatment, the most abundant were *rpoB* (*S. aureus* mutation), *gyrB* (*S. aureus* mutation), *tetA, mdtM*, *acrF*, and *macB* (0.48, 0.41, 0.41, 0.41, 0.27, and 0.27% respectively). Throughout the post-treatment, comparable to day 0 and the control sample, *rpoB* (*S. aureus* mutation*)* were the most abundant genes co-occurring with ARGs (1.38% of all the genes) followed by *gyrA* (*E. coli* mutation), *acrF*, and *tetQ* (0.54, 0.54, and 0.24%). Of all the ARGs, just *TEM-190* (0.27%), *QnrS1* (0.20%), *tetG* (0.13%), and *floR* (0.06%) were found close to a transposon (Tn3) in samples obtained during the antibiotic exposure phase and the first day of post-exposure (day 11). Before the antibiotic exposure, 1.26 and 0.84% of all ARGs were flanked by the MGEs phage integrases and *IS21* respectively. During the antibiotic exposure phase, 1.72, 1.51, 1.03, 0.69, 0.69, and 0.69% of all ARGs were flanked by the elements phage integrase, *IS21*, transposase, transposon (Tn3), *IS91*, and *IS3*. During the post-treatment, IS21 (1.27%) was also found flanking several genes.

**Table 1:** Percentage of ARGs co-occurring with MGEs before, during, and after antibiotic exposure

Day	Phage integrases	Transposon	Transposase	RteC	Resolvase	Total*
0	1.78 ± 0.69	0.00 ± 0.00	1.93 ± 0.53	0.20 ± 0.28	0.28 ± 0.39	4.19 ± 0.04
1	*0.96 ± 0.47*	*0.00 ± 0.00*	*3.52 ± 1.00*	*0.00 ± 0.00*	*0.18 ± 0.26*	*4.66 ± 1.72*
4	*1.36 ± 0.33*	*0.63 ± 0.45*	*4.40 ± 0.83*	*0.19 ± 0.26*	*0.34 ± 0.47*	*6.91 ± 1.03*
7	*2.21 ± 0.84*	*1.53 ± 0.12*	*6.09 ± 0.31*	*0.00 ± 0.00*	*0.95 ± 0.48*	*10.78 ± 0.57*
11	2.61 ± 2.61	0.00 ± 0.00	3.05 ± 3.05	0.00 ± 0.00	0.29 ± 0.29	5.95 ± 5.95
13	2.72 ± 1.63	0.00 ± 0.00	2.84 ± 0.83	0.41 ± 0.29	0.00 ± 0.00	5.96 ± 2.26
18	1.42 ± 0.56	0.00 ± 0.00	2.32 ± 0.76	0.00 ± 0.00	0.16 ± 0.23	3.91 ± 0.70
26	0.80 ± 0.57	0.00 ± 0.00	2.25 ± 2.09	0.19 ± 0.26	0.62 ± 0.47	3.87 ± 2.30
34	1.12 ± 1.59	0.00 ± 0.00	2.94 ± 2.52	0.00 ± 0.00	0.00 ± 0.00	4.07 ± 1.50
Control	0.93 ± 1.31	0.00 ± 0.00	0.82 ± 1.16	0.27 ± 0.38	0.40 ± 0.56	2.41 ± 1.29

Days under antibiotic treatment are presented in italics. ± indicates the S.D. Control was sampled at day 34

*Total includes ARGs co-occurring with more than one MGE

### Bacterial taxa harboring ARGs

Throughout the antibiotic exposure, the genus harboring most of the ARGs detected was *Bacteroides* (25.3% of all ARGs) despite its relatively low abundance, followed by *Citrobacter* (20.1%), *Klebsiella* (9.05%), *Plesiomonas* (8.29%), *Parabacteroides* (3.22%), and *Cetobacterium* (2.46%). Further, 9.12% members of *Enterobacteriaceae* could not be assigned to a specific genus (Additional file 1: Figure S10). Genera that increased their abundance due to the antibiotic exposure were linked to different ARGs, up to 63 found in *Citrobacter*, 52 in *Klebsiella*, 25 in *Plesiomonas*, 13 in *Cetobacterium*, and 9 found in *Parabacteroides*. Also, 49 in members of *Enterobacteriaceae* could not be assigned.

The most abundant genes associated with *Citrobacter* were *mexD*, *mexN*, and *emrD* (1.2, 0.75, and 0.61% respectively). Similarly, the most abundant genes for *Plesiomonas* were *mexW*, *mexQ*, and *rpoB* (*M. tuberculosis* mutation; 0.54, 0.48, and 0.34% respectively). After the antibiotic exposure treatment, *Plesiomonas* (2.33% of all ARGs) and *Cetobacterium* (1.19%) remained as one of the genera harboring the most abundant ARGs. Interestingly, *Bacteroides* was the only taxa associated with ARGs found before the antibiotic and constant during the exposure phase. In addition, most of the ARGs identified before, during and after antibiotic exposure phase was associated with this genus *(*25–57%), mostly the gene *mexQ* (9.05–22.91%).

## Discussion

### Potential mobility of antibiotic resistance genes

The effect of prophylactic, metaphylactic, and therapeutic administration of antibiotics on the gut bacterial communities of fish has received little attention so far. Instead, most of the studies have focused on the water columns and sediment of the farms applying aquaculture. We presume that the gut of the fish under antibiotic pressure is a perfect environment for the exchange of ARGs and MGEs. As predicted, we found that the abundance of ARGs and MGEs increased under antibiotic treatment. Furthermore, we observed a positive correlation between total ARGs and MGEs co-occurring with ARGs, showing a significant increase in potential mobilization of ARGs. This indicates that MGEs could be responsible for the prevalence of ARGs during the antibiotic pressure or a possible enrichment of taxa harboring ARGs co-occurring with MGEs. Similarly, it has been reported that tetracycline (*tet*) and sulfonamide (*sul1*) genes positively correlated to transposases in sediments from Baltic Sea fish farms and Chinese swine farms, respectively [6, 16]. These mobile elements were the most common flanking ARGs in our study. This is consistent with the finding of transposases as the most prevalent genes in nature [17]. It is known that they play an important role in bacterial evolution. They are involved in the mobility of genes and rearrangement of plasmids and chromosomes [18, 19]. Transposases members of the families *IS21* and *IS6* were the most abundant insertion sequences flanking ARGs during the antibiotic exposure. Interestingly, some members of *IS6* interact with transposons, and *IS21* has been reported to be associated with an increase of β-lactam resistance and to be involved in the mobility of ARGs coffering resistance to phenicols [19–21]. The fact that members of these families responded to florfenicol is an indicator of the potential exchange of genes. Besides, the family *IS21* was the most abundant insertion sequence found flanking ARGs before and after antibiotic treatment. This could indicate an important role of this element in the studied bacterial communities, possibly related to florfenicol pre-exposition.

Phage integrases seem to play an important role in the exchange of genetic material under antibiotic pressure. Their increase came along with the detection of several phages during the antibiotic treatment, mostly Enterophages. Recently, it was reported that viromes from non-human sources including freshwater and marine environments are reservoirs of ARGs [22]. Additionally, phages can transfer beneficial traits such as antibiotic resistance to neighboring cells [23]. In the present study, phage integrases were found flanking the gene *rpoB*, which encodes the β-subunit of bacterial RNA polymerase, and a few antibiotic efflux pumps. Similarly, it has been found that virome sequences from carbadox in-feed swine carry ATP-binding cassette (ABC) efflux pumps and their expression was enriched at least 10 times during the antibiotic treatment [24]. Also, it was shown that the presence of ciprofloxacin increases the mutation frequency of *rpoB* in *Escherichia coli* [25]. Moreover, polymerase mutants can enhance or diminish the SOS system response [26]. This is important because SOS response is involved in phage induction [27], which can explain that *rpoB* and other genes were found flanked by phage integrases in our samples. Our results indicating an increase in the abundance of phages, phage integrases, and ARGs flanked by phage integrases strengthen the idea of phage as vehicles of antibiotic resistance in the presence of antibiotics.

In addition, Tn3 transposon was detected exclusively during the antibiotic input flanking tetracycline, β-lactamase, fluoroquinolone, and florfenicol resistance genes in low abundance. Tn3 transposons commonly carry antimicrobial passenger genes, recruit mobile integrons, and promote the exchanges of gene cassettes [28, 29]. The enrichment of transposons seems to be mediated by the recruitment of different genes. That was the case for the Tn2 enrichment and dispersion during the phase of high aminopenicillin consumption during the 1960s and 1970s of the last century due to the recruitment of *bla*_TEM1a_ [30]. This could explain why this element was detected only during antibiotic exposure. Thus, the presence of *TEM-190*, *QnrS1*, *tetG*, and *floR* close to Tn3 can indicate that the use of florfenicol can promote the dispersion of transposons in aquaculture.

The rise of ARGs associated with plasmids carrying ARGs in our samples during the antibiotic exposure is a clear indication of potential mobility. Self-transmissible plasmids can promote horizontal gene transfer in an in vivo Zebrafish model without antibiotic pressure [31]. This suggests that aquatic animals can contribute to the dissemination of ARGs in water via conjugation. In addition, different bacterial isolates from the gut of fish have the in vivo potential to spread ARGs [32]. In our study, members of the family *Enterobacteriaceae* increased during the antibiotic exposure. The variability of plasmids able to facilitate antibiotic resistance in this bacterial family is high [33]. For example, the plasmid family’s lncFII and lncA/C highly occurred among typed resistance plasmids. Some taxa associated with these plasmids are *E. aerogenes*, *E. cloacae*, *E. coli*, *K. pneumoniae*, *S. enterica*, *S. marcescens*, *S. sonnei*, *C. freundii*, *C. koseri*, *K. oxytoca*, *P. mirabilis*, *P. stuartii*, and *S. marcescens* [33, 34]. *Citrobacter*, *Klebsiella*, and *Salmonella* were enriched during the antibiotic exposure in our study; this could be related to the presence of plasmids. For instance, all florfenicol resistance genes from our samples were identified as part of a plasmid, two of them associated with transposases, and one with Tn3. This finding indicates that florfenicol resistance and mobility in this environment could be mediated by plasmids. The origin of resistance to florfenicol has been under debate; it was initially detected in terrestrial bacteria associated with humans, but later it was found in a bacterium from aquaculture, which also indicates its mobility [35, 36]. Nowadays, this gene has been detected in plasmids isolated from humans and cows, co-occurring with different ARG as ceftriaxone and ceftiofur [37, 38]. The presence of *floR* in plasmid sequences, its emergence during antibiotic pressure, and its detection in terrestrial and marine environments represent a risk for the dissemination of antibiotic resistance.

### Enrichment of antibiotic resistance genes after antibiotic exposure

Aquaculture could be one of the main promoters of ARG enrichment in the environment [12]. Our study demonstrated that ARGs are enriched at least 4.5 times in the gut of the fish during antibiotic exposure. Similar results were described for pigs where more than 20 ARGs were enriched after the exposition with an antibiotic cocktail [39]. Similarly, to our study, the authors found that several resistance genes not related to the exposed antibiotic were also enriched. Those genes in our case were coding for resistance against multidrug, peptide, aminocoumarin, and tetracycline. Our findings also suggest that florfenicol could also co-select multi-resistance because of the increase in multiple efflux pump systems. Other study reported positive and negative associations between antimicrobial exposure and the number of antimicrobial resistant genes [40]. For example, macrolide promoted resistance to sulfonamide, lincomycin to macrolide, penicillin to tetracycline, and aminoglycoside to sulfonamide. This co-selection effect was also described for fish tanks. He et al. [41] showed that long periods of antibiotic input increases the diversity and abundance of specific ARGs. They found that tetracycline resistance genes are more easily inducible than sulfanilamide and β-lactamase resistance genes. Furthermore, the emergence of those genes was related to the antibiotic applied and the mix of different antibiotics increased the co-selection of genes. Florfenicol resistance can be produced by the genes *floR*, *pp-flo*, *fexA*, *flo*, *cfrC*, and *poxtA* [42]. Out of all these genes, only *floR* was detected in our fish gut samples during antibiotic input. However, we could not see a specific increase in genes coding for phenicol resistance class, which is associated with florfenicol. This could be associated with fish larvae rearing with florfenicol administration. Additionally, tetracycline resistance genes were enriched, for example, *tetA* and *tetG* were found flanked by MGEs during the antibiotic exposure. It seems that tetracycline resistance genes are one of the most common drug classes found in fish feces [6], fishmeal [7], and fish ponds [8]. However, florfenicol exposure in aquaculture tends to co-select mainly multidrug and peptide resistance genes.

In general, *mexQ*, *macB*, and other several efflux pump genes were the most abundant ARGs in the gut of the fish, also without the pressure of antibiotic. These genes are related to multidrug and macrolides resistance. A previous study reported that independent from the exposure with sulfonamide-trimethoprim, feces from fish harbored multidrug/efflux and macrolide/efflux resistance genes as well as tetracycline and chloramphenicol resistance genes [6]. Additionally, resistance genes of clinical relevance (e.g., β-lactams, fluoroquinolones, macrolides, and sulfonamides) have been detected in wild fish with no direct antibiotic exposition but closed to polluted sediments and water [43]. This could indicate that fish gut is a reservoir of ARGs and potential mobilization. An idea supported by the findings of Muziasari et al. [6], who described that fish feces contribute to the enrichment of antibiotic resistance genes in sediments. In that way, our data shows that this reservoir could potentially increase during antibiotic exposure in the fish gut and later be mobilized to other environments.

### Bacterial composition associated with ARGs

Bacteria from aquatic and terrestrial environments share several MGEs and ARGs [12], indicating a flow of genetic determinants between different environments. This represents a risk because of the possibility of multiresistant bacteria emerging from aquatic environments exposed to antibiotic as aquaculture farms. In this study, the antibiotic treatment promoted the emergence of different *Enterobacteriaceae* such as *Citrobacter*, *Klebsiella*, and *Plesiomonas* associated with ARGs. Members of these genera have been associated with nosocomial infections. For example, *Citrobacter* resistant to multiple β-lactamases [44, 45] and *Klebsiella* to β-lactamases, quinolones, and aminoglycosides [46] have been described*.* Additionally, strains from these species are known for harboring plasmids with different resistance mechanism [47]*.* In addition*, Citrobacter* was isolated from diseased fish and farm-rise catfish, carrying ARGs as *sulI*, *tetA*, *tetB*, and other tetracycline genes [48, 49]. This shows the importance of these bacteria in both aquatic and clinical environments. We found that most of the contigs identified as these bacteria carry genes coding for multiple multidrug efflux pumps. However, to a lower extent, *Citrobacter* and *Klebsiella* were associated with plasmid-mediated quinolone resistance and *Plesiomonas* to β*-lactamases*. Therefore, florfenicol not only enriches potentially pathogenic bacteria but also promotes resistance to different antibiotics. Equally important, the findings in this study indicate that bacterial community harboring ARGs from the gut of farmed *P. mesopotamicus* is mainly composed by the genus *Bacteroides*. However, members of the phylum *Proteobacteria* carrying ARGs were more abundant during the input of the antibiotic. The abundance of *Bacteroides* carrying ARGs during the different phases of the experiment can be explained by the dominance of the phylum *Bacteroidetes* in the gut of the animal. Also, *Bacteroides* are naturally resistant to aminoglycosides, and some strains carry genes that provide resistance to penicillin, cephalosporine, tetracycline, and macrolides [50]. In addition, it has been proven that the expression of efflux pumps of *Bacteroides fragilis* increased in response to oxidative and bile/bile salts stress [51]. Moreover, *Bacteroidetes* was identified as a potential host of tetracycline resistance genes in an effluent of coastal aquaculture in South Korea [52]. In the present study, *Bacteroides* were associated with multiple resistance antibiotic efflux and target modification genes (*rpoB* and *gyrB*). It has been proven that mutation in *gyrA* and *gyrB* of *B. fragilis* confer resistance to fluoroquinolone [53]. In this way, *Bacteroides* could be an important reservoir of antibiotic resistance because of its dominant abundance in the intestines of *P. mesopotamicus.* The detection of common bacteria from water and sediments and the emergence of pathogens during the exposition of antibiotic in fish increased the risk for public health and ARGs dispersion. Finally, our data and analyses provide a base for continuing the exploration of the mobilization of ARGs in the environment. Additionally, emerging technologies as long read-sequencing could be used as a next step to evaluate the synteny of different genes obtained from fish gut samples before during and after the antibiotic exposition. This could elucidate the potential transfer of genes in aquaculture.

## Conclusion

Overall, our findings suggest that while the prophylactic use of antibiotics in fish aquaculture intends to contribute to its management, it might actually introduce several risks. The antibiotic pressure increases the bacterial stress response, the number of ARGs and MGEs, the co-occurrence of these elements, and enrichment of *Enterobacteriaceae* members in the gut of the animal. We see an increased potential for the mobilization of ARGs during the antibiotic exposure. Additionally, the increased number of both plasmids and phages could facilitate the horizontal transfer of the mobilized ARGs. The fact that important nosocomial pathogens carrying several ARGs are enriched is alarming. Furthermore, the association of the most dominant taxa with multiple antibiotic efflux pumps and target alteration genes could be a signal of antibiotic resistance dissemination due to aquaculture practices. However, we cannot differentiate between the increased potential for ARG mobilization caused by the observed shift in the bacterial community and/or actual horizontal gene transfer and acquisition of ARG-MGE elements. Finally, most of the studies related to ARGs in aquaculture had focused on sediments and water samples, and few of them on the real-time transfers of the genes under antibiotic pressure. In this way, our study highlights the risk of using in-feed antibiotic during aquaculture production due to the potential increase of ARG mobilization and dispersion.

## Methods

### Experimental setting

All experiments and protocols using *P. mesopotamicus* were approved by the Ethics Committee for Animal Experimentation of the University of Campinas, Brazil (protocol #2015-39). One hundred fifty male juvenile animals, with an average weight of approximately 724 g (Additional file 1: Figure S1), were kept in ten 0.8-m^3^ plastic tanks and adapted at 25.8 °C for 30 days in a continuously aerated loop system. During that time, the animals were fed with a commercial non-medicated feed (Nutripeixe; Purina do Brasil Ltda., Paulínia, Sao Paulo, Brazil), twice per day at 9 a.m. and 5 p.m. After the adaptation phase, the animals were randomly distributed between 10 tanks, which were operated under the same conditions as described above, resulting in 15 animals per tank. Animals in eight of the 10 tanks were fed for the period of 10 consecutive days with medicated feed, resulting in a dose of 9.7 mg florfenicol per kg and day. The FDA approved the use of a dose of 10–15 mg/kg body weight/day for 10 consecutive days of Aquaflor, which is mainly based on florfenicol, in recirculating aquaculture system [54]. The remaining tanks served as a control and day 0 and animals received non-medicated feed throughout. After the treatment phase, all animals received again the non-medicated feed for the duration of 24 days. Nine sampling time points were chosen: day 0 (pre-exposure phase); days 1, 4, and 7 (exposure phase); and days 11, 13, 18, 26, and 34 (post-exposure phase). The control tank was sampled at day 34. Data generated during the experiment was also used for a depletion study and estimation of withdrawal period for florfenicol in *Piaractus mesopotamicus*. The exposure phase was defined as the period when fish received the antimicrobial (day 1 to day 10). On day 11, the fish received only non-medicated feed and this phase is considered the depletion phase. Residues of florfenicol and florfenicol amine in the skin and muscle of the fish were 941, 388, 201, and 137 ng g^−1^ at days 11, 13, and 16 (not included in this study) respectively. After day 16, the antibiotic concentration was under detection limit. One tank was randomly chosen for each sampling time point, and three fish were collected and treated as true replicates for the metagenomics analysis. Animals were sacrificed using a solution containing 500 mg L^−1^ benzocaine. The gut samples were collected at least 4 h after the fish feeding in the morning period. The abdominal cavity was dissected and the gastrointestinal tract was aseptically and immediately collected. Fecal material was removed by gentle squeezing the distal section of the intestine (S3 to rectum) [55, 56]. Samples were transferred to a sterile tube and immediately snap frozen on dry ice and stored at − 80 °C for subsequent DNA extraction. Further details on the experimental design and estimation of withdrawal period can be found in [57].

### DNA extraction and metagenomics library preparation

Metagenomic DNA was extracted using the QIAmp fast DNA Stool Mini Kit (Qiagen, Valencia, CA, USA) following the manufacturer’s instructions. Only two DNA extractions from sample day 11 yielded enough DNA for the preparation of metagenomics libraries, and obtained results from the corresponding samples were highly variable. DNA concentrations were measured using Quant-It™ PicoGreen® dsDNA Assay Kit (ThermoFisher Scientific, MA, USA) and a spectrofluorometer (SpectraMax Gemini EM microplate reader Molecular Devices, LLC, USA). DNA purity check was assessed spectrophotometrically (Nano Drop 1000, ThermoFisher Scientific, USA). In total ~ 100 ng of DNA per sample was sheared using an E220 Focused-ultrasonicator (Covaris® Inc., MA, USA) targeting 500 bp fragments following Covaris’s instructions. Metagenomic libraries were constructed using NEBNext® Ultra™ DNA Library Prep Kit for Illumina®. Dual indexing was done using the kit NEBNext® Multiplex Oligos for Illumina® (Dual index primers set 1, New England BioLabs, UK). Purification and size selection was performed based on Agencourt® AMPure® XP (Beckman-Coulter, MA, USA). Libraries inserts ranged between 500 and 700 bp were evaluated using a Fragment Analyzer™ (Advanced Analytical, IA, USA). One sample with sterile water was used as a control for the metagenomics library preparation and sequencing. Libraries quantification were performed using Quant-It™ PicoGreen® dsDNA Assay Kit and sequenced on an Illumina MiSeq (Illumina, CA, USA) using the paired-end mode (2 × 300 bp).

### Quality control and general bioinformatic analysis

Adapters and primers were removed from raw reads using Adapterremoval v.2.1 [58]. Nucleotides with quality values less than 15 were trimmed and sequences shorter than 50 bp discarded. PhiX internal Illumina control and host DNA contamination was filtered using Deconseq v.0.4 [59]. A database was created with the reference genomes of *Pygocentrus nattereri* (BioProject: PRJNA331139) and *Astyanax mexicanus* (BioProject: PRJNA237016) for a Deconseq-decontamination step due to the absence of *P. mesopotamicus* genome. Clean reads were taxonomically classified by Kaiju v1.4.5 [60] in a greedy mode allowing five substitutions. Only reads assigned to Bacteria and phages were used for further analysis. Nonpareil v2.4 [61] was used to estimate the metagenomes’ coverage and calculate Nonpareil diversity index, which is a proxy for describing the complexity of the bacterial community. Orthologous groups (OGs) were predicted using the eggNOG database [62] and Diamond v.0.8 [63] using the “more-sensitive” mode. Orthologous groups predicted were mapped against the COG database [64], and best hits were selected. The data was normalized by the total of hits obtained.

### Antibiotic resistance genes and mobile genetic elements prediction

Total cleaned reads were assembled using metaSPADES v 3.10 [65] with a maximum k-mer size of 127; for downstream analysis, only contigs larger than 500 bp were retained. Protein-coding genes were predicted using prodigal v2.6.3 with default parameters using the “meta” mode for metagenomic data. Contigs with two or more open reading frames (ORF) predicted were used for further analysis.

ARGs were detected with Resistance Gene Identifier v3.1.1 and “The Comprehensive Antibiotic Resistance Database” (CARD) [42] using “strict” bitscore cut-offs. Contigs harboring ARGs were taxonomically classified using Kaiju v1.4.5 and analyzed for the possibility of a plasmid origin using PlasFlow v.1.0 [66]. Here, only contigs larger than 1 kb were used for the prediction of plasmid sequences (Additional file 1: Figure S10).

MGEs homologs were searched using the PFAM 31 [67] and TnpPred [68] databases through HMMER v3.1b2 [69]. Hits with a maximum 1 × 10^−5^ e-value were retained, and the best hit per read was used for further analysis. MGEs were grouped into six groups based on identified MGEs: phage integrases, transposons (transposases related to a specific transposon), transposases, RteC (related to tetracycline transposon), resolvases, and others. Position and co-occurrence of ARGs and MGEs were analyzed using in-home scripts. Additionally, co-occurrence of genes was curated manually. Co-occurrence was considered positive if an antibiotic resistance gene was found within ten open reading frames from upstream or downstream a mobile genetic element gene. Details about this can be found in (Additional file 2).

### Statistical analysis and visualization

Statistical analysis and plots were created using R v3.3.1., SigmaPlot v12., and LefSe-Galaxy v1.0. Linear discriminant analysis (LDA) was used to determine biomarkers for every treatment using LEfSe [70]. Only for this analysis, data was normalized as reads per million per sample; for the pairwise Wilcoxon test, an alpha value of 0.05 and 3.5 as the logarithmic LDA score threshold for discriminative features was used. Likelihood ratio test (LRT; DESeq2) [71] was used to analyze for differences in terms of functional annotation between the samples. LRT compares a full model vs a reduce model. In our case, the reduced model consisted of “all sampling points − 1”. All significantly different orthologous groups between treatments (LRT *P* < 0.5 and abundance > 0.001%) were used for preparing a ternary plot. The dissimilarity between the taxonomical, functional, and ARG bacterial structure of the day 0 and post-antibiotic phase was explored using the Bray-Curtis dissimilarity measure and represented using a Non-metric Multidimensional Scaling (NMDS) plot. Additionally, dissimilarity was tested using Adonis test (permutation = 999). Multivariate homogeneity of group dispersions was also tested. Samples from the antibiotic phases and day 11 (post-antibiotic) were excluded to observe the differences. Vegan v.2.4-2 package was used for this analysis. Differences in the relative abundance of ARGs, MGE, and fold change between drug classes were evaluated using robust one-way ANOVA and robust post hoc Rand Wilcox’s based on trimmed means and percentile bootstrap [72]. Here, the *t1way* (α = 0.05, and trimmed mean = 5%) and *mcppb20* (bootstrap = 2000 and trimmed mean = 20%) functions implemented by Wilcox were utilized for the analysis. Differences in the relative abundance of ARGs and MGEs between days 0 and 7 were explored using the Jonckheere–Terpstra test implemented in the *clinfun* R-package [73]. This test evaluates the significance of ARGs’ and MGEs’ enrichment each day over the time of the experiment considering, as an alternative to medians homogeneity, that the relative abundance of ARGs and MGEs is increasing every day between day 0 and day 7 in our experimental setup. Relative abundance was calculated using the number of ORF predicted. Moreover, the correlation of the log-transformed relative abundance of MGEs flanking ARGs was evaluated by a robust Spearman’s correlation implemented by Wilcox as the function *bootTau()* (bootstrap = 2000). All the Wilcox’s functions can be found in [74]. Total ribosomal protein L1 and L12 genes and the same genes co-occurring with MGEs before, during, and after antibiotic treatment were used as a control for the correlation of MGE flanking genes (Additional file 1: Figure S8).

## Additional files

Additional file 1: **Figure S1.** Variation of weight of the sampled fish before, during and after exposure to florfenicol. **Figure S2.** Coverage/number of raw reads (A) and Nonpareil index diversity (B) from the bacterial community of *Piaractus mesopotamicus* before during and after antibiotic exposure. The index indicates the complexity of the bacterial community in terms of “Sequencing space”. Higher values indicate higher diversity. **Figure S3.** Effect of florfenicol on the relative abundance of the families (A) and genus (B) of the bacterial community of *Piaractus mesopotamicus* before, during, and after antibiotic exposure*.* **Figure S4.** Taxonomic (A), functional (B), and ARGs (C) structure of the gut bacterial community of *Piaractus mesopotamicu* before (day 0) and after antibiotic exposure (days 13, 18, 26, 34). **Figure S5.** Effect of the antibiotic florfenicol on the relative abundance of ARGs (A) and MGEs (B) of the gut bacterial community of *Piaractus mesopotamicus* before, during and after antibiotic treatment. **Figure S6.** Fold changes of different drug classes after the antibiotic treatment. **Figure S7.** Antibiotic resistance genes from plasmid origins. **Figure S8.** Correlation of total ribosomal protein L1 (A) and L12 (B) and the same genes co-occurring with MGEs genes before, during and after antibiotic exposure. **Figure S9.** The 30 Most abundant ARGs flanked by MGEs in the gut of *Piaractus mesopotamicus* before, during, and after antibiotic exposure. **Figure S10.** The eight most abundant genus (contigs) harboring ARGs from gut samples of *Piaractus mesopotamicus* before, during and after the antibiotic exposure. **Table S1.** Bacterial functional shift in the gut of *P. mesopotamicus* before, during, and after antibiotic exposure. (DOC 5958 kb)

Additional file 2: The file is the script used to find the genes co-occurring with the ARGs detected. (RB 4 kb)

## Abbreviations

ARG: Antibiotic resistance gene
MGE: Mobile genetic element
