# Supplementary material for: Oral administration of antibiotics increased the potential mobility of bacterial resistance genes in the gut of the fish Piaractus mesopotamicus
Source: Microbiome. 2019 Feb 18;7:24. doi: 10.1186/s40168-019-0632-7 (PMC6378726; doi:10.1186/s40168-019-0632-7)
Supplement: Supplementary file 1 — Figure S1. Variation of weight of the sampled fish before, during and after exposure to florfenicol. Figure S2. Coverage/number of raw reads (A) and Nonpareil index diversity (B) from the bacterial community of Piaractus mesopotamicus before during and after antibiotic exposure. The index indicates the complexity of the bacterial community in terms of “Sequencing space”. Higher values indicate higher diversity. Figure S3. Effect of florfenicol on the relative abundance of the families (A) and genus (B) of the bacterial community of Piaractus mesopotamicus before, during, and after antibiotic exposure. Figure S4. Taxonomic (A), functional (B), and ARGs (C) structure of the gut bacterial community of Piaractus mesopotamicu before (day 0) and after antibiotic exposure (days 13, 18, 26, 34). Figure S5. Effect of the antibiotic florfenicol on the relative abundance of ARGs (A) and MGEs (B) of the gut bacterial community of Piaractus mesopotamicus before, during and after antibiotic treatment. Figure S6. Fold changes of different drug classes after the antibiotic treatment. Figure S7. Antibiotic resistance genes from plasmid origins. Figure S8. Correlation of total ribosomal protein L1 (A) and L12 (B) and the same genes co-occurring with MGEs genes before, during and after antibiotic exposure. Figure S9. The 30 Most abundant ARGs flanked by MGEs in the gut of Piaractus mesopotamicus before, during, and after antibiotic exposure. Figure S10. The eight most abundant genus (contigs) harboring ARGs from gut samples of Piaractus mesopotamicus before, during and after the antibiotic exposure. Table S1. Bacterial functional shift in the gut of P. mesopotamicus before, during, and after antibiotic exposure. (DOC 5958 kb) [file 40168_2019_632_MOESM1_ESM.doc]

**Supplementary information**

**Oral administration of antibiotics increased the potential mobility of bacterial resistance genes in the gut of the fish *Piaractus mesopotamicus***

Johan S. Sáenz^a^, Tamires Valim Marques^b^, Rafael Simões Coelho Barone^c^, José Eurico Possebon Cyrino^c^, Susanne Kublik^a^, Joseph Nesme^a,d^, Michael Schloter^a,e^*, Susanne Rath^b^ & Gisle Vestergaard^a,d^

^a^ Comparative Microbiome Analysis, Helmholtz Zentrum München, 85764 Oberschleissheim, Germany

^b^ Institute of Chemistry, University of Campinas, Campinas, Brazil

Section of Microbiology, Department of Biology, University of Copenhagen, 2100 Copenhagen, Denmark

^c^ Departamento de Zootecnia, Escola Superior de Agricultura Luiz de Queiroz, University of São Paulo, Piracicaba, Brazil

^d^ Section of Microbiology, Department of Biology, University of Copenhagen, 2100 Copenhagen, Denmark

^e^ ZIEL -Institute for Food & Health; Technical University of Munich, 85354 Freising, Germany

* Corresponding author: schloter@helmholtz-muenchen.de

**Supplementary figures and tables**

**Figure S1.** Variation of weight of the sampled fish before, during and after exposure to florfenicol.

**A**

**B****
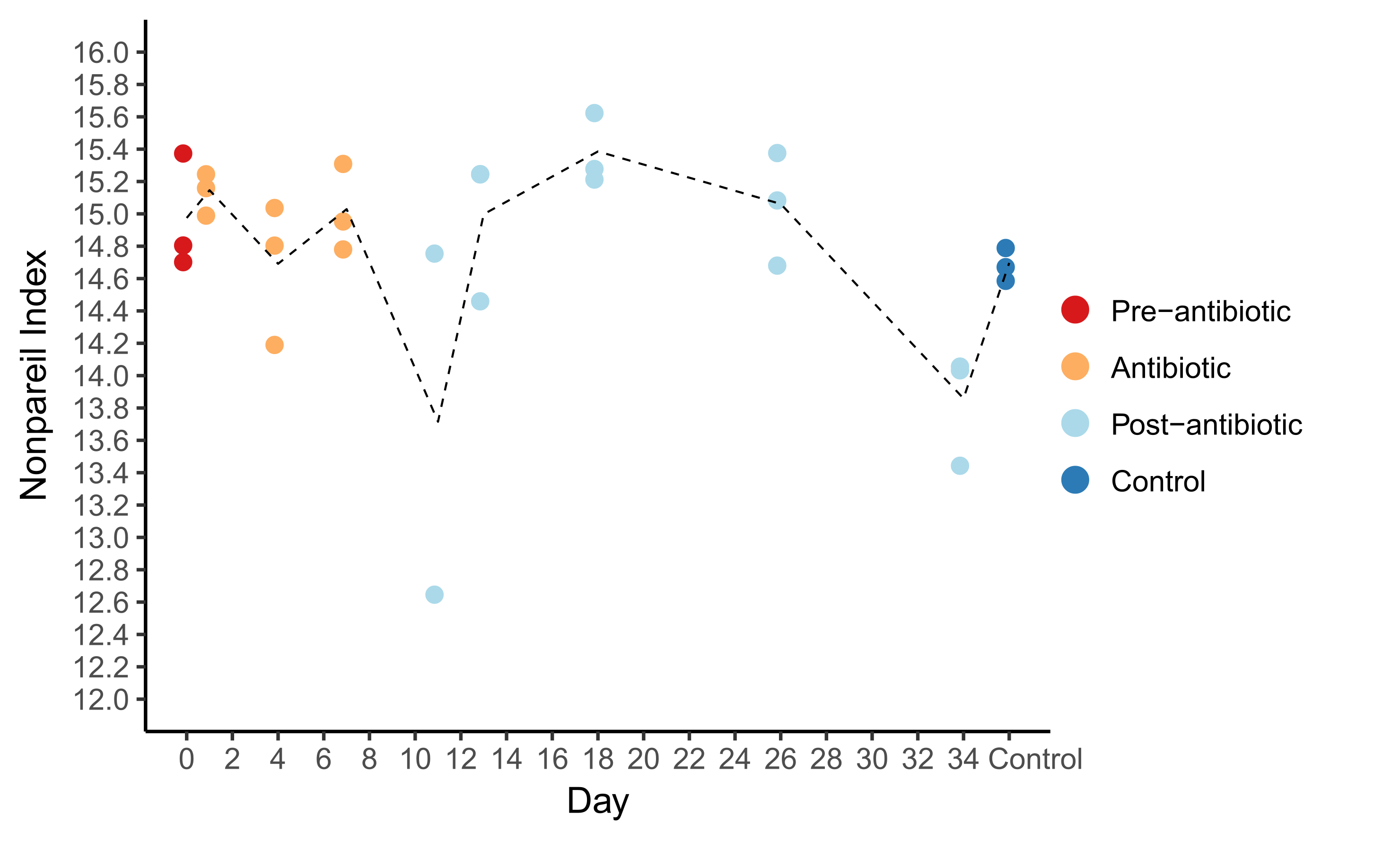
**

**Figure S2.** Coverage/number of raw reads (A) and Nonpareil index diversity (B) from the bacterial community of *Piaractus mesopotamicus* before during and after antibiotic exposure. The index indicates the complexity of the bacterial community in terms of “Sequencing space”. Higher values indicate higher diversity.


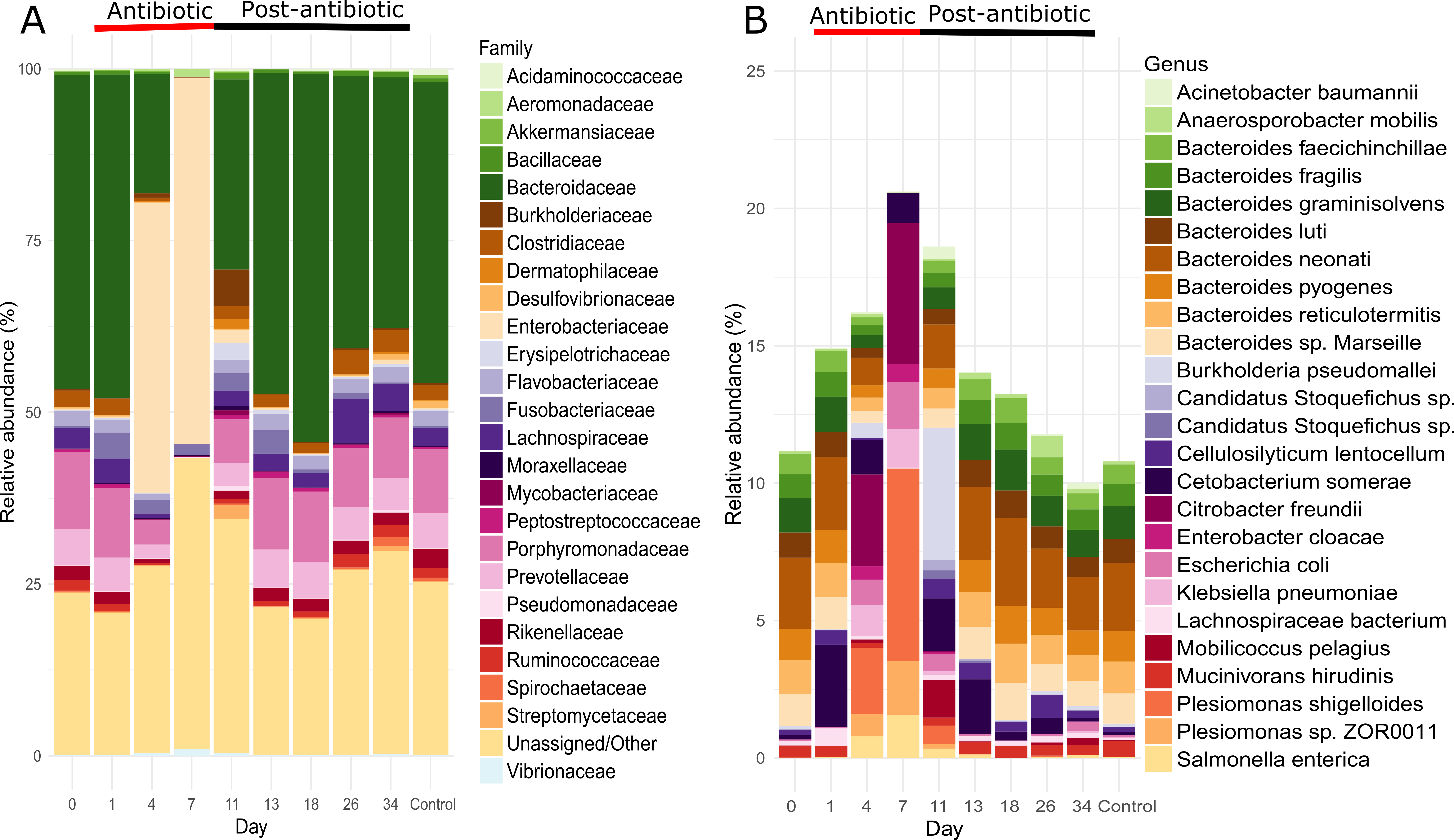


**Figure S3.** Effect of florfenicol on the relative abundance of the families (A) and genus (B) of the bacterial community of *Piaractus mesopotamicus* before, during and after antibiotic exposure***.*** Day 0 (pre-exposure), 1, 4, 7 (exposure/florfenicol), 11, 13, 18, 26 and 34 (Post-exposure). Additionally, it is shown the control sample taken at day 34 of a tank that did not receive antibiotic during the experiment. The 30 most abundant families and genus are shown.


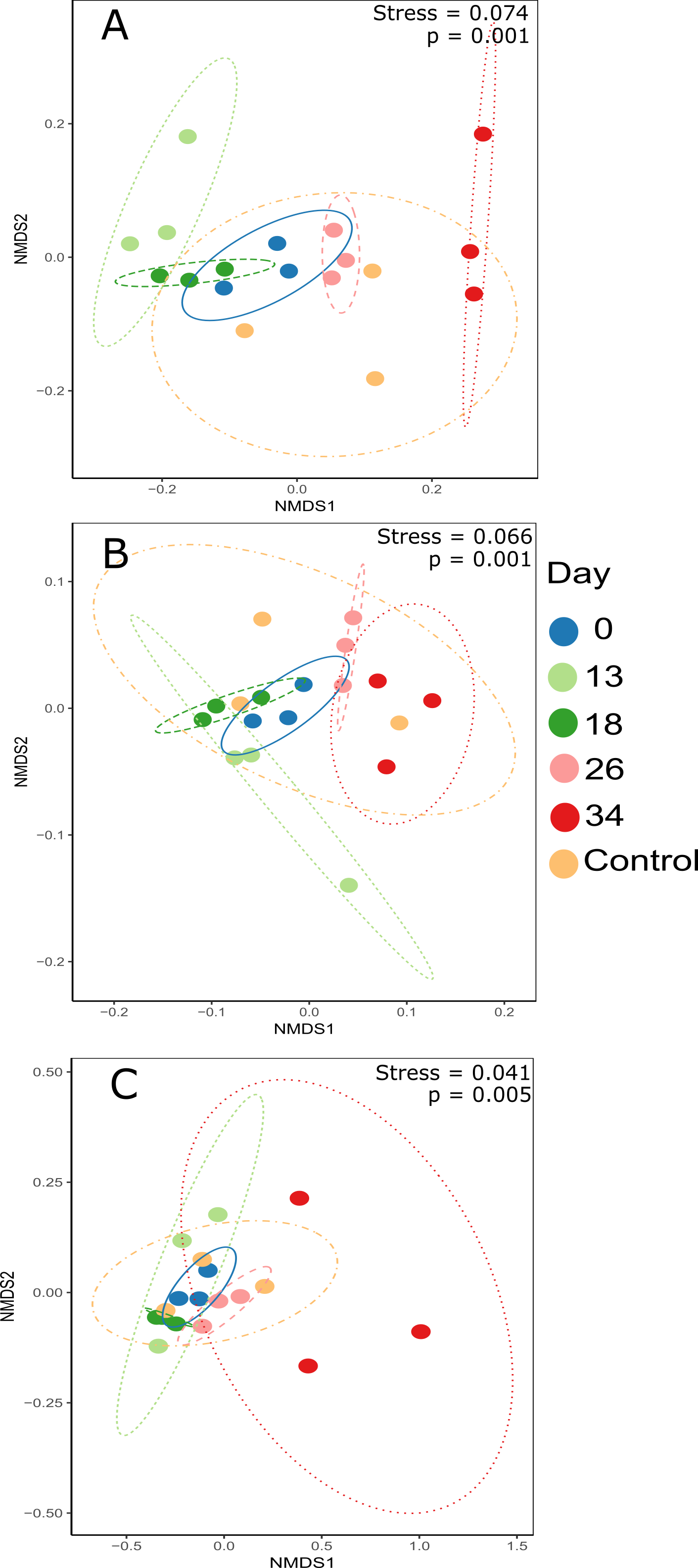


**Figure S4.** Taxonomic (A), functional (B), and ARGs (C) structure of the gut bacterial community of *Piaractus mesopotamicu* before (day 0) and after antibiotic exposure (day 13, 18, 26, 34). Additionally, it is shown the control sample taken at day 34 of a tank that did not receive antibiotic during the experiment. Dissimilarity was calculated using the Bray-Curtis dissimilarity measure and represented using a Non-metric Multidimensional Scaling (NMDS) plot. Additionally, dissimilarity was tested using The Analysis of Similarity (ANOSIM, permutation = 999). Multivariate homogeneity of group dispersions was also tested.

**
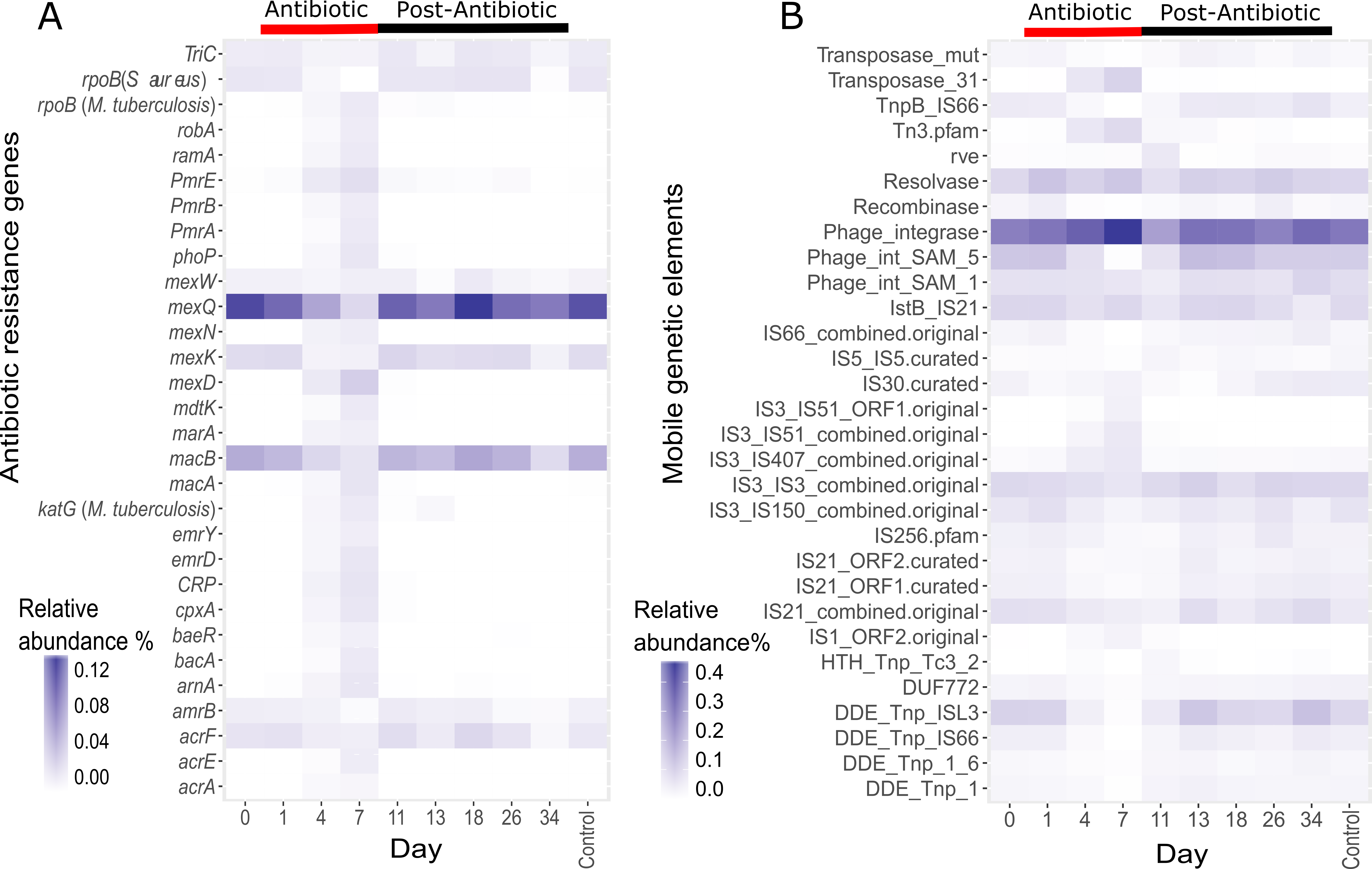
**

**Figure S5.** Effect of the antibiotic florfenicol on the relative abundance of ARGs (A) and MGEs (B) of the gut bacterial community of *Piaractus mesopotamicus* before, during and after antibiotic treatment. Day 0 (pre-exposure), 1, 4, 7 (exposure/florfenicol), 11, 13, 18 , 26 and 34 (Post-exposure). Additionally, it is shown the control sample taken at day 34 of a tank that did not receive antibiotic during the experiment. The 30 most abundant ARGs and MGEs are shown


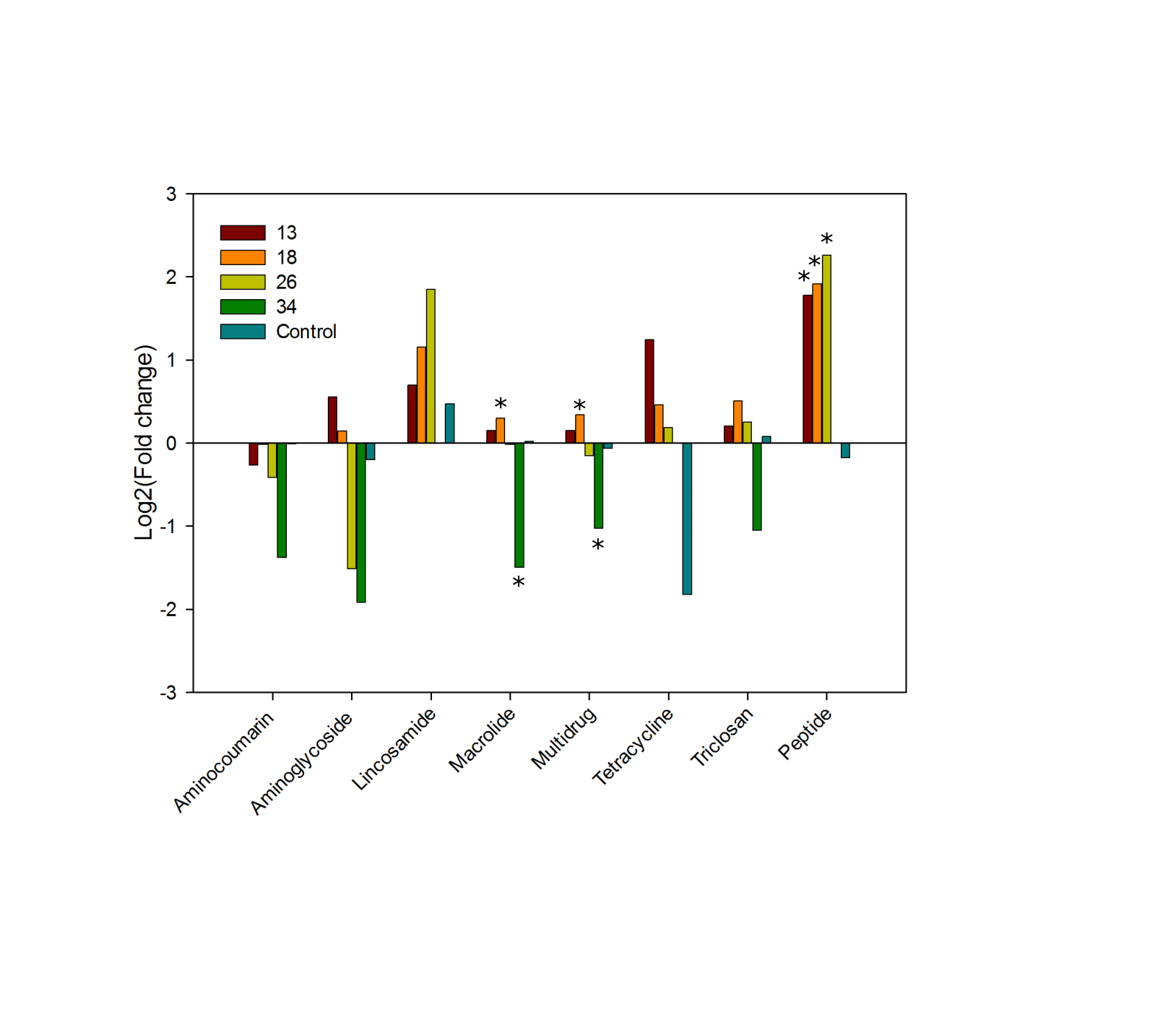


**Figure S6.** Fold changes of different drug classes after the antibiotic treatment. Day 0 was compared against day 13, 18 26 and 34. Additionally, control sample was included. Log2 fold change between drug classes were evaluated using robust one-way ANOVA and robust post hoc Rand Wilcox’s based on trimmed means and percentile bootstrap. * p < 0.05.

**Figure S7.** Antibiotic resistance genes from plasmid origins. Relative abundance of contigs (>1 kb) identified as a plasmid sequence (dark blue) and plasmid sequences carrying ARGs (light blue). Day 0 (pre-exposure), 1, 4, 7 (exposure/florfenicol), 11, 13, 18, 26 and 34 (Post-exposure). Additionally, it is shown the control sample taken at day 34 of a tank that did not receive antibiotic during the whole experiment.

**A**


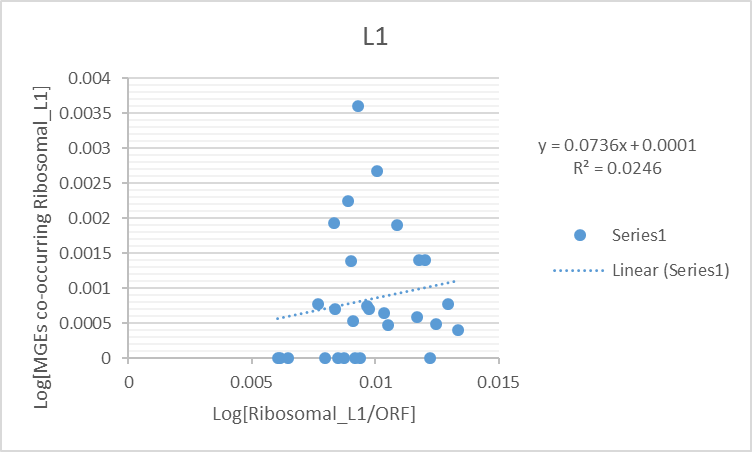


**B**


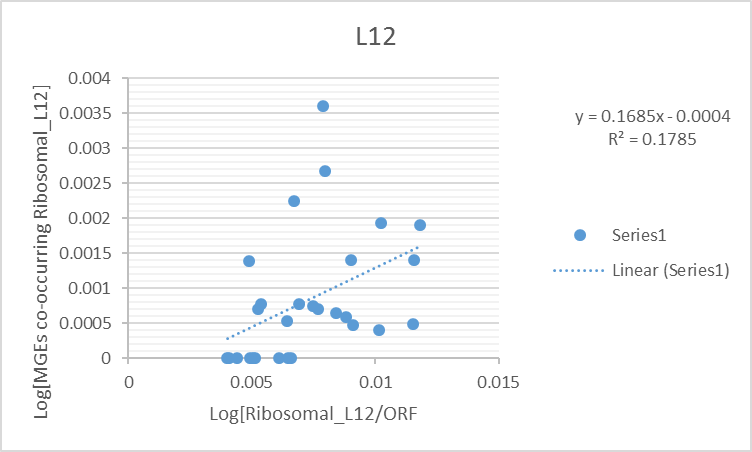


**Figure S8.** Correlation of total ribosomal protein L1 (A) and L12 (B) and the same genes co-occurring with MGEs genes before, during and after antibiotic exposure. Data used as a control of ARGs and MGEs association. **L1** : (Spearman´s correlation = 0.24, S = 2775.5, P = 0.2178), **L12**: (Spearman´s correlation = 0.54, S = 1652, P = 0.002)


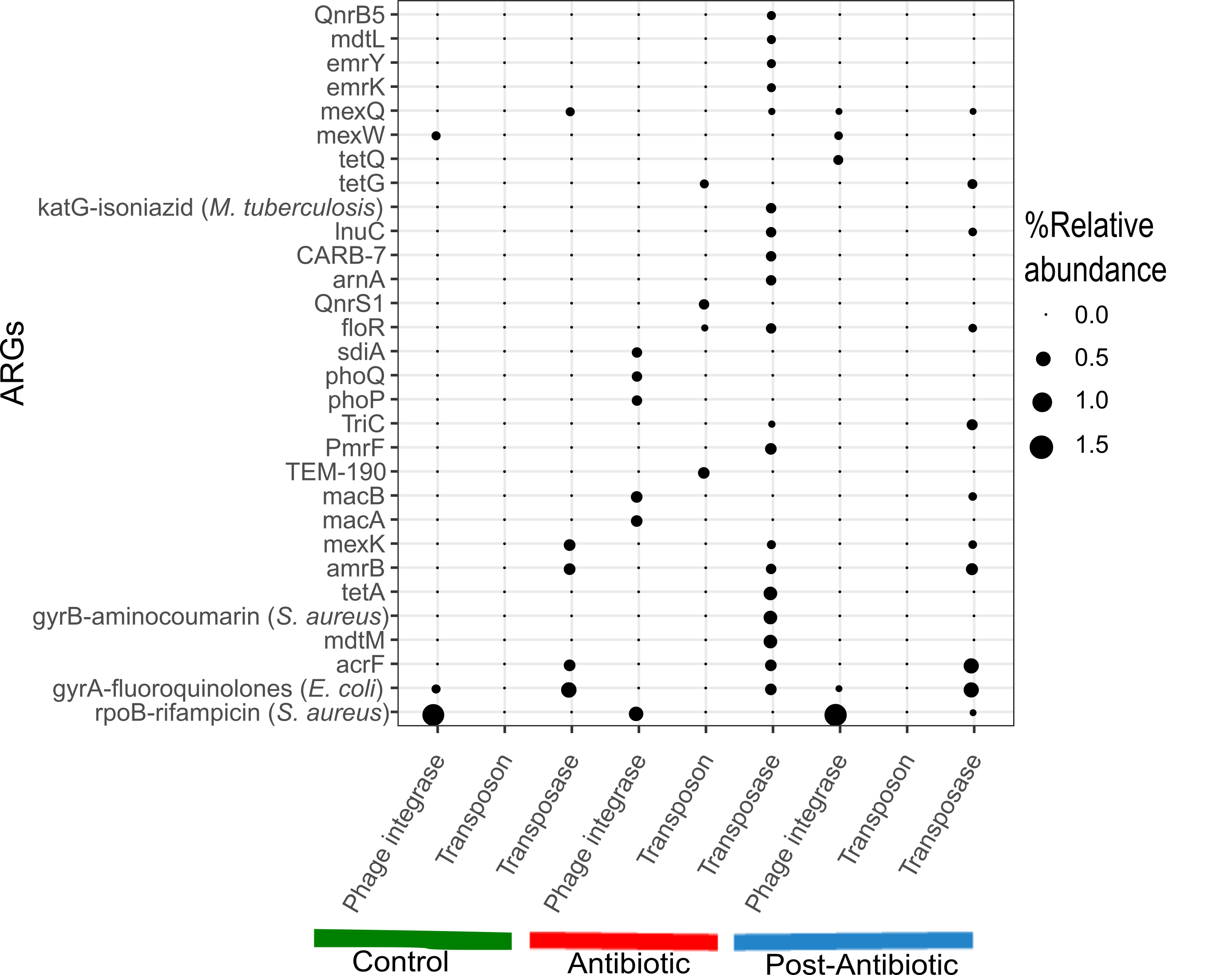


**Figure S9.** The 30 Most abundant ARGs flanked by MGEs in the gut of *Piaractus mesopotamicus* before, during and after antibiotic exposure. Flanking element was found maximum 10 ORFs further from the ARGs. The figure is divided in three sections, 1) control (day 0 and control), 2) antibiotic/exposure (day 1, 4 and 7) and 3) post-antibiotic/post-exposure (day 11, 13, 18, 26 and 34).**
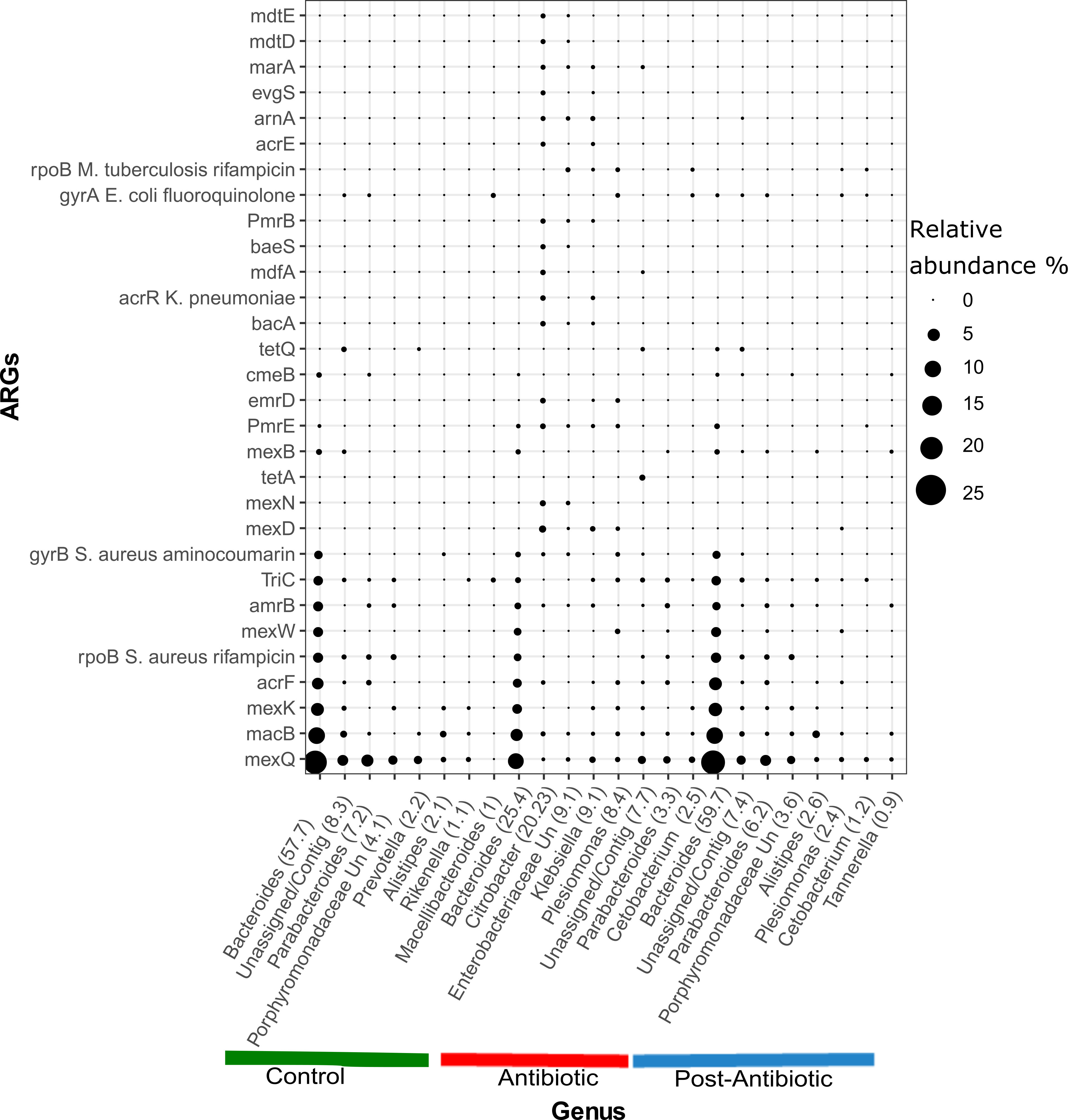
Figure S10.** The eight most abundant genus (contigs) harboring ARGs from gut samples of *Piaractus mesopotamicus* before, during and after the antibiotic exposure. The figure is divided in three sections, 1) control (day 0 and control), 2) antibiotic/exposure (day 1, 4 and 7) and 3) post-antibiotic/post-exposure (day 11, 13, 18, 26 and 34). Number between parentheses indicates the percentage of total ARGs linked to the genus

**Table S1.** Bacterial functional shift in the gut of *P. mesopotamicus* before, during and after antibiotic exposure

|  | **Pre-exposure** | **Antibiotic exposure** | | | **Post-exposure** | | | | | **Control** | **p-val** |
| --- | --- | --- | --- | --- | --- | --- | --- | --- | --- | --- | --- |
| **Day** | 0 | 1 | 4 | 7 | 11 | 13 | 18 | 26 | 34 |
| **RNA processing and modification** | 0.00036 | 0.00010 | 0.01262 | 0.01833 | 0.06718 | 0.00027 | 0.00009 | 0.00156 | 0.00328 | 0.00111 | **1.81E-21** |
| **Chromatin Structure and dynamics** | 0.00270 | 0.00328 | 0.00554 | 0.00196 | 0.11986 | 0.00322 | 0.00185 | 0.00601 | 0.00936 | 0.00788 | **4.88E-12** |
| **Cell cycle control and mitosis** | 0.86086 | 0.87774 | 0.86665 | 0.91744 | 1.10683 | 0.86398 | 0.85224 | 0.89497 | 0.86678 | 0.86427 | **0.007454** |
| **Replication and repair** | 7.24171 | 7.34151 | 6.09230 | 6.62537 | 7.10613 | 7.25413 | 7.33507 | 7.56655 | 7.70343 | 7.58495 | **0.000546** |
| **Signal Transduction** | 3.66773 | 3.79367 | 4.16465 | 4.01460 | 5.22098 | 3.90090 | 3.56358 | 3.71584 | 4.09204 | 3.75847 | **0.010763** |
| **Intracellular trafficking and secretion** | 1.14575 | 1.11368 | 1.77497 | 2.29495 | 1.74158 | 1.10682 | 1.13970 | 1.25413 | 1.23278 | 1.20834 | **1.12E-12** |
| **Transcription** | 2.88403 | 2.99750 | 5.03468 | 5.87953 | 3.14788 | 3.00819 | 2.74972 | 3.12853 | 3.22007 | 2.97796 | **1.20E-16** |
| **Secondary Structure** | 0.53022 | 0.52491 | 1.12561 | 1.22488 | 0.54773 | 0.52086 | 0.52617 | 0.53869 | 0.54258 | 0.55914 | **5.16E-25** |
| **Energy production and conversion** | 5.93564 | 5.77996 | 7.03175 | 7.27665 | 5.37646 | 5.58766 | 6.03760 | 5.94952 | 5.70984 | 5.98652 | **1.79E-08** |
| **Post-translational modification** | 3.26726 | 3.19893 | 3.35920 | 3.45542 | 3.67900 | 3.25754 | 3.20961 | 3.29243 | 3.31297 | 3.29803 | 0.478117 |
| **Inorganic ion transport and metabolism** | 8.03445 | 7.84763 | 7.67335 | 6.96078 | 5.48286 | 8.09654 | 7.94146 | 7.43913 | 7.37011 | 7.61797 | 0.062691 |
| **Translation** | 5.38721 | 5.17130 | 4.32974 | 4.12239 | 5.02791 | 5.16811 | 5.31321 | 5.79692 | 5.51577 | 5.67773 | **4.96E-08** |
| **Cytoskeleton** | 0.00848 | 0.00506 | 0.02209 | 0.00028 | 0.58411 | 0.00444 | 0.00455 | 0.01703 | 0.03442 | 0.01112 | NA |
| **Amino Acid metabolism and transport** | 7.98701 | 7.77839 | 8.73985 | 9.46905 | 6.90837 | 7.66797 | 7.97864 | 8.02278 | 7.71210 | 7.98314 | **3.04E-05** |
| **Carbohydrate metabolism and transport** | 10.02663 | 10.35502 | 9.36557 | 8.57148 | 7.64144 | 10.15008 | 10.56781 | 9.90629 | 9.37571 | 9.91669 | 0.073204 |
| **Coenzyme metabolism** | 2.61013 | 2.60571 | 2.72097 | 2.99066 | 2.19765 | 2.55184 | 2.74840 | 2.51884 | 2.39851 | 2.64055 | **5.64E-07** |
| **Nucleotide metabolism and transport** | 2.67997 | 2.59216 | 2.33799 | 2.36710 | 2.57196 | 2.61351 | 2.64972 | 2.83530 | 2.71232 | 2.72523 | **3.06E-06** |
| **Cell wall/membrane/envelop biogenesis** | 6.69959 | 6.68918 | 6.78470 | 6.52408 | 5.44304 | 6.95158 | 6.63488 | 6.59205 | 6.69363 | 6.79667 | 0.094776 |
| **Lipid metabolism** | 1.89164 | 1.79430 | 1.98677 | 2.01404 | 2.00863 | 1.82973 | 1.83325 | 1.86782 | 1.91146 | 1.92890 | 0.194879 |
| **Cell motility** | 0.18289 | 0.18786 | 0.71956 | 1.15133 | 0.23442 | 0.13513 | 0.13969 | 0.31334 | 0.26414 | 0.20701 | **7.47E-28** |
| **Function Unknown** | 26.76970 | 27.08863 | 24.07814 | 22.41499 | 31.57244 | 27.13969 | 26.61129 | 26.05356 | 26.99044 | 25.99996 | **0.003807** |
| **Extracellular structures** | 0.00062 | 0.00148 | 0.01076 | 0.01007 | 0.01349 | 0.00080 | 0.00094 | 0.00158 | 0.00157 | 0.00062 | **7.79E-07** |
| **Defense mechanisms** | 2.18541 | 2.25197 | 1.76254 | 1.69465 | 2.20000 | 2.18701 | 2.16052 | 2.28714 | 2.32667 | 2.24772 | **5.07E-07** |

The table shows the relative abundance of different Cluster orthologous groups (COGs) and the significance (p-val) of the change of the abundance over time. Significance was detected using the Likelihood ratio test (LRT), *P* < 0.05 DeSeq2. Highlighted numbers indicate significant differences. Data for 3 phases is shown, pre-exposure, antibiotic exposure and post-exposure. NA: not determined
